# Patterns of African and Asian admixture in the Afrikaner population of South Africa

**DOI:** 10.1186/s12915-020-0746-1

**Published:** 2020-02-24

**Authors:** N. Hollfelder, J. C. Erasmus, R. Hammaren, M. Vicente, M. Jakobsson, J. M. Greeff, C. M. Schlebusch

**Affiliations:** 1 Human Evolution, Department of Organismal Biology, Uppsala University, Norbyvägen 18C, SE-752 36 Uppsala, Sweden; 2 Department of Biochemistry, Genetics and Microbiology, University of Pretoria, Pretoria, 0002 South Africa; 3 Science for Life Laboratory, Uppsala University, Norbyvägen 18C, SE-752 36 Uppsala, Sweden; 4 Palaeo-Research Institute, University of Johannesburg, P.O. Box 524, Auckland Park, 2006 South Africa

**Keywords:** Afrikaner, South Africa, Admixture, Slave trade, Colonial times

## Abstract

**Background:**

The Afrikaner population of South Africa is the descendants of European colonists who started to colonize the Cape of Good Hope in the 1600s. In the early days of the colony, mixed unions between European males and non-European females gave rise to admixed children who later became incorporated into either the Afrikaner or the Coloured populations of South Africa. Differences in ancestry, social class, culture, sex ratio and geographic structure led to distinct and characteristic admixture patterns in the Afrikaner and Coloured populations. The Afrikaner population has a predominant European composition, whereas the Coloured population has more diverse ancestries. Genealogical records previously estimated the contribution of non-Europeans into the Afrikaners to be between 5.5 and 7.2%.

**Results:**

To investigate the genetic ancestry of the Afrikaner population today (11–13 generations after initial colonization), we genotyped approximately five million genome-wide markers in 77 Afrikaner individuals and compared their genotypes to populations across the world to determine parental source populations and admixture proportions. We found that the majority of Afrikaner ancestry (average 95.3%) came from European populations (specifically northwestern European populations), but that almost all Afrikaners had admixture from non-Europeans. The non-European admixture originated mostly from people who were brought to South Africa as slaves and, to a lesser extent, from local Khoe-San groups. Furthermore, despite a potentially small founding population, there is no sign of a recent bottleneck in the Afrikaner compared to other European populations. Admixture amongst diverse groups from Europe and elsewhere during early colonial times might have counterbalanced the effects of a small founding population.

**Conclusions:**

While Afrikaners have an ancestry predominantly from northwestern Europe, non-European admixture signals are ubiquitous in the Afrikaner population. Interesting patterns and similarities could be observed between genealogical predictions and our genetic inferences. Afrikaners today have comparable inbreeding levels to current-day European populations.

## Background

The seventeenth-century European colonization of the southern tip of Africa resulted in the influx of two groups of people, European colonists and slaves. The subsequent admixture between these external groups and the local southern African Khoe-San populations resulted in two admixed populations—the Afrikaner population and the Coloured population of South Africa [1] (in this article, we use the term “Coloured” following the current-day continued use of the term as self-identification [2]).

While both the Afrikaner and Coloured populations have ancestry from many populations from different continents, the ancestry proportions differ substantially between the groups. The admixture proportions of these populations do not reflect the historical local census sizes of the parental populations (Additional file 1: Supplementary Text) [3–31]; rather, ancestry, social class, culture, sex ratio and geographic structure affected admixture patterns [5–7, 32, 33].

The most dominant contribution to the Afrikaner population came from European immigrants ([9, 30, 31] and Additional file 1: Supplementary Text), whereas the Coloured population has more diverse ancestries [34–43]. The colonization of southern Africa started in 1652 when the Dutch East India Company (DEIC) established a refreshment station at the Cape of Good Hope (Cape Town today). In 1657, employees of the DEIC were released from their services to start farming [7]. This group, numbering 142 adults and children in 1658, continued to grow due to high fecundity (almost 3% per annum) and continued immigration, and their descendants became the Afrikaners (Additional file 1: Supplementary Text, [7]). Two other major sources of immigrants were 156 French Huguenots that arrived in 1688 and an unknown number of German labourers and soldiers that were financially marginalized [7]. Estimates, based on genealogical research, vary but Dutch, German and French respectively contributed 34–37%, 27–34% and 13–26% ([9, 30, 31] and Additional file 1: Supplementary Text).

While the DEIC did not encourage admixture with local populations and slaves, the strongly male-biased ratio of immigrants led to mixed-ancestry unions [32], especially between European males and non-European females [33]. The offspring from these unions were frequently absorbed into the Afrikaner population [9]. As time progressed, relationships between Europeans and non-Europeans became more infrequent [9], and as early as 1685, marriages between Europeans and non-Europeans were outlawed (marriages to admixed individuals, with some European ancestry, were still allowed though) [33]. In early colonial times, mixed marriages were more acceptable than later on, and due to the population’s fast growth rate, early unions likely contributed exponentially more to the Afrikaner population. Elphick and Shell [32] distinguish two admixture patterns in Afrikaners based on historical records—in Cape Town and the surrounding area admixture was predominantly between European men and female slaves or former slaves, and in the outlying areas between European pastoralist frontier farmers (“trekboere”) and Khoe-San women.

Admixture with slaves (and former slaves) resulted from informal as well as formal associations [32]. The church recorded many marriages between Europeans and manumitted slaves [9, 33]. It is unclear what the input of informal relationships into the Afrikaner gene pool was, as the outcome of these relationships and the population affiliation of the resulting offspring were not recorded. One source of informal liaisons was the slave lodge that served as a brothel for 1 h a day for passing sailors and other European men [13, 32]. This practice was so extensive that many children in the slave lodge clearly had European fathers (3/4 in 1671; 44/92 in 1685; 29/61 of school children and 23/38 children younger than 3 years in 1693 [3]). Many women that married at the Cape during the early years used the toponym “van de Kaap” (meaning from the Cape) which may indicate a locally born slave. European men also sometimes had a “voorkind” (meaning “before child”) with a slave in the household before they got married to a European woman [32]. These children could also have been absorbed into the Afrikaner population (as opposed to becoming part of the Coloured population).

To understand the characteristics of the genetic contributions that slaves made, it is necessary to know from where and when they came to Cape Town and see that in the light of European male partner choices. Shell [4] claimed that from 1658 to 1807, roughly a quarter of the slaves in the Cape colony came from Africa, Madagascar, South Asia and Southeast Asia each. Slave trade in the Cape was stopped in 1807, and slavery as such was stopped in 1834. Worden [11, 12] estimated that more slaves came from Asia, specifically South Asia, and fewer from Madagascar and Africa (Additional file 1: Supplementary Text). Nevertheless, we do not expect an exact reflection of these ratios in Afrikaners. European men had a clear preference for Asian and locally born slaves over African and Malagasy women [32]. Despite only two ships, containing West African slaves, that moored at the Cape in 1685 [10], we can expect the West African per capita contribution to exceed later arrivals because the fast population growth rate meant earlier contributions benefitted more from the exponential growth.

The “trekboere” were European farmers who followed a nomadic lifestyle in harsh conditions along the frontier. Informal unions with Khoe-San women were more frequent amongst the “trekboere”, but it is unclear if children from these relationships were absorbed into the Coloured and/or Afrikaner community [7, 32]. Poor record keeping and a reduced presence of the church on the frontier meant that recorded information is incomplete for this section of the population. In the Cape, formal unions between European men and Khoe-San women were very unusual with only one known example [3].

By using church records, genealogists calculated the contribution of non-Europeans to be between 5.5 and 7.2% ([9, 30, 31] and Additional file 1: Supplementary Text). These estimates may be biased because the registers (a) only reflect the Christian fraction of the population, (b) were less complete at the frontier where admixture may have been more frequent, (c) could be incorrectly pieced together from church records and (d) list people of unknown heritage, such as “van de Kaap”. In addition, records may be incorrect or unrecorded for children born out of wedlock. Populations that would have been excluded were a substantial Muslim community amongst manumitted slaves [32], a small Chinese population resulting from exiles and banned political prisoners [44, 45] and the indigenous Khoe-San who were not partial to the Christian religion [32]. The presence of the Coloured population compounded these difficulties as genes may have exchanged between the Coloured and Afrikaner populations.

In order to clarify the patterns of ancestry and admixture fractions in current-day Afrikaners, we compared genome-wide genotype data from 77 Afrikaners to comparative data from potential donor populations and tried to pinpoint the best possible sources of the admixture and the fractions of admixture from these groups.

## Results

### Population structure and admixture

We generated filtered genotype data for 77 Afrikaner individuals (the “Materials and methods” section) and merged the Afrikaner data with comparative data to create a dataset containing 1747 individuals from 33 populations and 2,182,606 SNPs. We used this merged dataset to conduct population structure analysis and to infer population summary statistics.

**Fig. 1:**
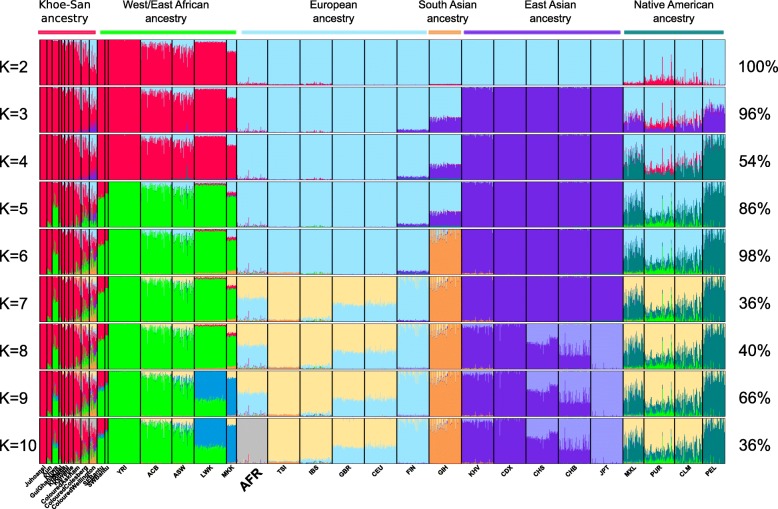
Admixture analysis of Afrikaners and comparative data. The numbers of allowed clusters are shown on the left and the statistical support on the right. Abbreviations: YRI Yoruba from Nigeria, ACB and ASW African American, LWK Luhya from Kenya, MKK Maasai from Kenya, AFR Afrikaner from South Africa, TSI Tuscan from Italy, IBS Iberian from Spain, GBR British from Great Britain, CEU Northwest European ancestry from Utah, FIN Finnish, KHV Vietnamese, CDX Dai from China, CHS Han from southern China, CHB Han from Beijing, JPT Japanese, MXL Mexican, PUR Puerto Rican, CLM Colombian, PEL Peruvian

In the population structure analysis (Fig. 1) [46], Afrikaners cluster with non-Africans (*K* = 2 to *K* = 9) and specifically Europeans (*K* = 3 to *K* = 9) before receiving their own cluster at *K* = 10. From *K* = 7 onwards, northern and southern Europeans cluster separately, with Finnish forming one cluster (light blue) and southern Europeans (Tuscans and Iberians) the other cluster (light yellow). British (GBR) and Utah residents of northwestern European descent (CEU) appear midway between the Finnish and southern European clusters. Afrikaners contain significantly more northern European (blue) ancestry component compared to CEU and GBR (*P* < 0.00001, Mann-Whitney *U* test). The specific percentage of cluster assignments of Afrikaner individuals at *K* = 6 and *K* = 9, and the population averages assigned to each cluster, are given in Additional file 1: Table S1 and S2.

**Fig. 2:**
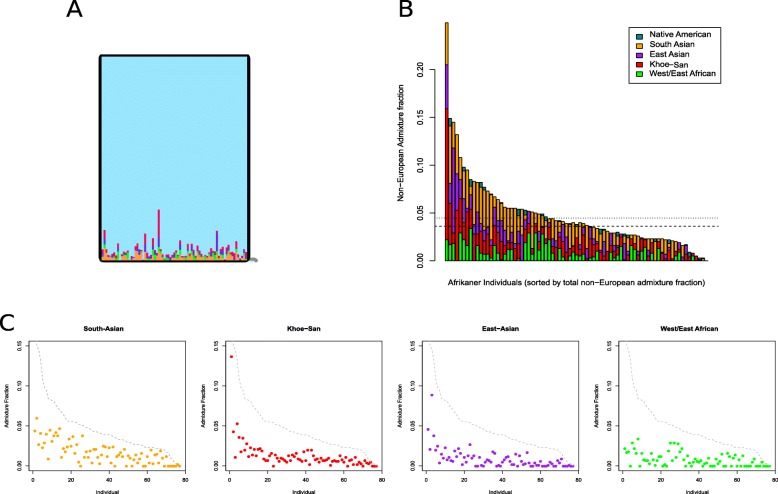
Admixture proportions of the Afrikaner at *K* = 6. **a** Magnification of the Afrikaner population in the ADMIXTURE analyses. **b** Non-European admixture fraction in the Afrikaner, sorted by total non-European admixture fraction. Dotted lines indicate the mean (top line) and median (bottom line). **c** Individual non-European admixture fractions sorted by total non-European admixture fraction (grey line)

Assuming six clusters (*K* = 6), where the major geographical ancestries are discernible, i.e. aboriginal southern African (Khoe-San), West/East African, European, East Asian, South Asian and Native American, the level of admixture from these ancestries can be distinguished in the Afrikaners (Additional file 1: Table S1 and Fig. 2). In addition to European ancestry (mean of 95.3% SD 3.8%—blue cluster), Afrikaners have noticeable levels of ancestry from South Asians (1.7%—orange cluster), Khoe-San (1.3%—red cluster), East Asians (0.9%—purple cluster) and West/East Africans (0.8%—green cluster), and very low levels from Native Americans (0.1%). The small fraction from Native Americans likely stems from common ancestry between Native Americans and Europeans and from European admixture into Native Americans. The total amount of non-European ancestry, at the *K* = 6 level, is 4.8% (SD 3.8%) of which 2.1% are African ancestry and 2.7% Asian and Native American ancestry. The individual with the most non-European admixture had 24.9% non-European admixture, and only a single Afrikaner individual (out of 77) had no evidence of non-European admixture (Additional file 1: Table S1). Amongst the 77 Afrikaners investigated, 6.5% had above 10% non-European admixture, 27.3% between 5 and 10%, 59.7% between 1 and 5% and 6.5% below 1%. The fractions of admixture from the different non-European groups in Afrikaners (at *K* = 6) are generally correlated to each other (Additional file 1: Figure S1), except for the West/East African admixture fractions.

At *K* = 9 (before Afrikaners form their own cluster at *K* = 10), additional inferences can be made regarding Japanese vs. Chinese ancestry, East vs. West African ancestry and northern vs. southern European ancestry (Additional file 1: Table S2). Southern and northern European ancestry is almost equal in the Afrikaners but northern European ancestry is elevated compared to CEU and GBR. Similarly, it seems that Afrikaners received East Asian ancestry from Chinese rather than Japanese individuals and slightly more West African ancestry than East African ancestry. These specific affinities were tested using f4 tests (Additional file 1: Table S3), and results supported the closer affinity to West Africans vs. East Africans, while the closer affinity to Chinese vs. Japanese was not supported.

Compared to the Afrikaners, the Coloured populations have more diverse origins. At *K* = 6, the Cape Coloured population from Wellington (within the region of the original Cape colony) had the following ancestry fractions: 30.1% Khoe-San, 24% European, 10.5% East Asian, 19.7% South Asian, 15.6% West/East African and 0.2% Native American (Fig. 1). The Coloured populations whom today are living further from the original Cape colony had different admixture patterns with less Asian and more Khoe-San contribution than the Cape Coloured: Colesberg Coloured (48.6% Khoe-San, 20% European, 2.9% East Asian, 6.7% South Asian, 21.6% West/East African, 0.2% Native American) and Askham Coloured (76.9% Khoe-San, 11.1% European, 0.9% East Asian, 3.9% South Asian, 7.2% West/East African, 0% Native American).

**Fig. 3:**
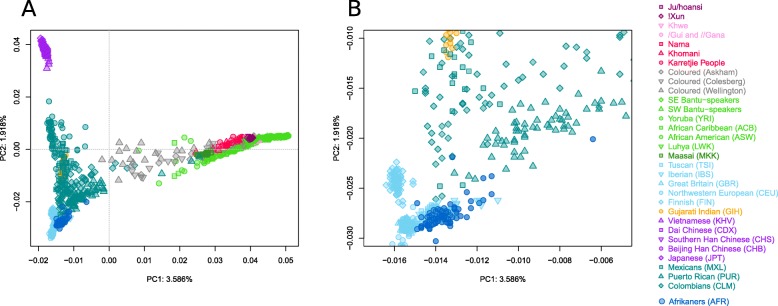
Principal component analyses showing PC1 and PC2. **a** Full figure. **b** A zoom-in on the Afrikaner (dark blue) population

In a principal component analysis (PCA) (Fig. 3 and Additional file 1: Figure S2), the first principal component (PC1) explains 3.6% of the variation in the dataset and distinguish Africans from non-Africans (right to left). PC2 explains 1.9% of the variation in the dataset and distinguishes Europeans from East Asians (top to bottom). The distribution of Afrikaners along PC1 and PC2 suggests both African and Asian admixture. Compared to northern Europeans (CEU and GBR), Afrikaners seem to have more African and East Asian admixture. From the PCA, it appears that most of the Afrikaner group have non-European ancestry at comparable levels to Iberians and Tuscans (IBS and TSI); however, certain Afrikaner individuals show greater levels of both African and Asian ancestry (Fig. 3).

To look for the most likely sources of admixture in the Afrikaners within the comparative dataset, we did formal tests of admixture (f3, [47]) between all pairs of comparative populations each time specifying (two) potential parental sources for the Afrikaner population. Results were sorted ascendingly according to *Z* scores (low *Z* scores indicate significant admixture), and *Z* scores are visualized in a figure to aid interpretation (Additional file 1: Figure S3); parental populations are coloured according to the regional population. It is clear that when only two populations are considered to be parental sources the most likely sources are always a European and either a Khoe-San or a West African population (combination of blue and red or blue and green labels in Additional file 1: Figure S3). Subsequently, the Coloured population can also be used to model a parental population in combination with Europeans (blue and grey labels). We also fixed a European source (CEU) to show the best African source, and an African source (≠Khomani) to show the best European source (Additional file 1: Figure S4). The best African sources appear to be Khoe-San populations and the best European sources, the CEU and GBR. This method is however limited by the fact that only two parental sources can be tested at one time and might not be the best tests when multiple parental groups admixed into a population, as is the case for the Afrikaner population.

Alleles that are shared privately between combinations of the Afrikaner population with one comparative population (Additional file 1: Figure S5) show that the Afrikaners share most private alleles with the CEU population, which makes them a better parental source than the other European populations. The ≠Khomani share the most private alleles with the Afrikaner out of all Khoe-San populations, indicating that southern San contributed to the Afrikaner population rather than northern San groups. The most shared private alleles of the Afrikaner with Asian populations can be found in the GIH (Gujarati Indian) followed by the CHB (Chinese) and JPT (Japanese). Regarding the West African fraction, Afrikaners share more private alleles with the Niger-Congo-speaking Yoruba (YRI) from Nigeria, intermediate levels with east African Bantu-speaking groups, Luhya (LWK) from Kenya and the lowest levels with southeastern Bantu speakers from South Africa (Additional file 1: Figure S6). This supports admixture from West African slaves and East African Bantu-speaking slaves rather than from southern African Bantu speakers into the Afrikaner population. Since the CEU and YRI populations were reference populations in the original design of Illumina SNP arrays, there is a potential effect of ascertainment bias and results should be interpreted with caution.

For finer scale resolution of the European and the Asian components in the Afrikaners, the dataset analysed above was furthermore merged, respectively, with the POPRES dataset [48, 49] and various datasets containing populations from East, South and Southeast Asia. These additional European and Asian comparative datasets had much lower SNP densities, but they contained many more comparative populations and were used for fine-scale resolution of European and Asian components in Afrikaners.

The Afrikaner individuals were projected on a PCA, constructed based on European variation present in the POPRES dataset (Additional file 1: Figure S7). Afrikaner individuals seem to group within western European variation. They are grouped in-between the French and German clusters on the PC plot. When principal components were summarized by population averages and standard deviations, they seem to be grouping closest to Swiss German, Swiss French and Belgian populations. This positioning could also be explained as an intermediate position between German, Dutch and French variation that link to each other in a clinal pattern [48, 49].

When analysing Afrikaners with a dataset enriched for Asian populations, it appears that the largest contributing Asian component is from India (Additional file 1: Figure S8). The orange component in Additional file 1: Figure S8 is the most prominent admixed component from Asian groups, and this component is specifically associated with Indo-European-speaking Indian groups, i.e. Khatri, Gujarati Brahmin, West Bengal Brahmin, and Maratha; and Dravidian speaking Iyer [50].

### Dating of admixture

We dated the time of admixture in the Afrikaners using a linkage disequilibrium (LD) decay method (“Materials and methods” section, Additional file 1: Table S4) [47]. Admixture from different founder groups could not be distinguished from one another, and only one admixture event was inferred, dated to 9.3 generations ago. The best parental populations were northwestern European groups (CEU, GBR and FIN) and Khoe-San groups (Ju/’hoansi and !Xun).

### Patterns of selection and allele frequency differences in the Afrikaner

We scanned Afrikaner genomes for patterns of allele frequency variations compared to comparative European source populations (CEU and GBR), by doing a locus-specific branch length (LSBL) analysis. Regions where allele frequencies were differentiated compared to the CEU and GBR population were plotted in a genome-wide Manhattan plot (Additional file 1: Figure S9). The top 5 peaks are listed and described in Additional file 1: Table S5. Amongst the top 5 peaks, four peaks had genes directly associated with the peak and the other peak was 18 kb upstream of a gene. Three of the five associated genes were protein coding with described functions and two genes were RNA genes with less known functions (Additional file 1: Table S5). The *SPECC1* gene associated with the top peak has strong expression in the testis and shows high similarity to a human sperm antigen gene (OMIM entry 608793). The gene associated with the second highest LSBL peak (*STK39*) has a role in the cellular stress response pathway and shows ubiquitous expression, with the most abundant expression in the brain and pancreas. The *ERF* gene associated with peak 3 is thought to belong to an oncogene family and play a role in embryonic development and cell proliferation.

We also analysed the Afrikaner data for genome-wide signals of selection by scanning for regions with extended haplotype homozygosity compared to other haplotypes within the same population (iHS scans) and compared to haplotypes in a comparative population (XP-EHH scans). For XP-EHH scans, Afrikaners were compared to the CEU population. Additional file 1: Figure S10 shows the genome-wide Manhattan plot of selection scan results for iHS and XP-EHH. Several peaks that might indicate signals of selection in the Afrikaner group were observed. The top 5 peaks in each scan are listed in Additional file 1: Table S6 with descriptive information. From the top 5 iHS peaks, only one had a gene directly associated with the peak; the gene *FGF2* is a fibroblast growth factor with a variety of functions and was previously associated with cholesterol levels. The XP-EHH results were clearer and three out of the five top peaks were directly associated with genes: *CCBE1*—a gene previously associated with lymphatic disease, *ACTG2*—an enteric smooth muscle actin gene previously associated with intestinal diseases and *SUCLG2*—encoding a succinate-CoA ligase, previously associated with glucose and fat metabolism. Interestingly, the Afrikaner group does not show the strong adaptation signals at the lactase persistence region on chromosome 2 and MHC region on chromosome 6, which are strong and well-known signals for the CEU group. Although the CEU group has significantly more of the lactase persistence associated allele (rs4988235-T) (71% in CEU vs. 52% in the AFR, *p* = 0.000434), their predicted lactase persistence phenotypic status, based on homozygote and heterozygote counts combined, is not significantly different (91% in CEU vs. 83% in AFR, *p* = 0.126514).

### Estimation of bottleneck effects

**Fig. 4:**
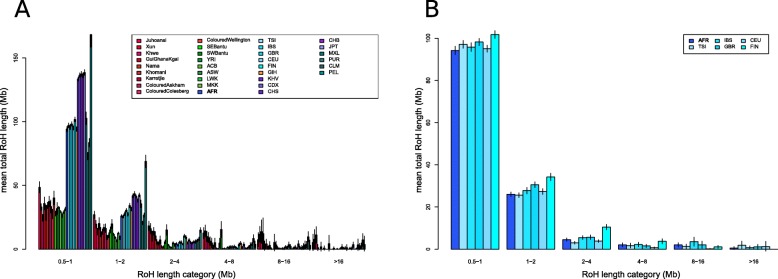
**a** Runs of homozygosity. Populations are coloured according to the regional group. **b** Magnification of runs of homozygosity for European populations and the Afrikaner

Runs of homozygosity were calculated for each individual of the dataset. Depending on their length, runs of homozygosity are informative of historic population size or recent inbreeding in populations [51]. While we see striking differences between continental groups (Fig. 4), there is no strong difference between the Afrikaner and other European populations, except for the Finish population that appears to have had a smaller historic effective population size (Fig. 4). The results were not noticeably affected by the low amounts of non-European admixture into the Afrikaners and when admixed fragments were masked out of Afrikaner genomes, similar results were obtained (Additional file 1: Figure S11).

## Discussion

Genealogical records suggest that Afrikaners have their main ancestry components from Europeans (Dutch, German and French) and estimate the non-European contributions to the Afrikaner to be between 5.5 and 7.2% ([9, 31] and Additional file 1: Supplementary Text). Our genetic study that included 77 Afrikaners inferred a slightly lower non-European contribution than predicted by genealogical studies. From population structure analyses, we saw that Afrikaners have their main ancestry component (95.3%) from European populations. The European component is a more northwestern (than southern or eastern) European component (Fig. 1 and Additional file 1: Figure S8), which is in agreement with genealogical records of most ancestry coming from Dutch and German (61–71%), intermediate from French (13–26%), with much smaller fractions from other European groups ([9] and Additional file 1: Supplementary Text). Of note, Afrikaners group separately from populations from the UK (Additional file 1: Figure S7) despite the fact that the Cape was a British colony from 1806 onwards. This confirms the relatively small contributions from British people to the Afrikaner population as predicted by genealogical records [9].

The non-European fraction in Afrikaners was estimated to be 4.7% on average (Additional file 1: Table S1). More of the non-European admixture fraction appeared to have come from people who were brought to the Cape as slaves (3.4%) during colonial times than from local Khoe-San people (1.3%). Indeed historical records of the early Cape Colony record more instances of unions between European men and slaves or former slaves than to local Khoe-San women [32]. Only one example of a Khoe-San-European union in the Cape colony is known. A local Khoekhoe woman from the Goringhaicona group, Eva (or Krotoa) van de Kaap, was an interpreter and ambassador between the colonists and Khoekhoe people and married Pieter Van Meerhof in 1664 [3, 52]. Since unions between Khoe-San women and the frontier farmers were thought to be more frequent, it may account for the 1.3% observed admixture in the Afrikaner population. The 1.3% observed Khoe-San ancestry calculates to 26.6 Khoe-San women out of 2048 ancestors 11 generations ago. However, we know that one Afrikaner had for example only 299 ancestors in colonial times [30] because many Afrikaner ancestors enter pedigrees multiple times [9, 30, 53]. These 26.6 Khoe-San women that contributed to the average Afrikaner should thus not be seen as 26 separate women (i.e. the same woman could have contributed many times). The Khoe-San admixture component is the most ubiquitous non-European admixture component and only 6 out of the 77 Afrikaners had no Khoe-San ancestry (Additional file 1: Table S1).

South and East Asians contribute cumulatively to an average of 2.6% of the Afrikaner ancestry (53.2 out of 2048 ancestors 11 generations ago). Elphick and Shell [32] noted that European men more often mixed with Asian and locally born slaves than African and Malagasy women. Although many other additional factors might have played a role in the resultant current-day Afrikaner admixture fractions, the genetic admixture fractions of South and East Asians were higher in current-day Afrikaners than Khoe-San fractions (1.3%) and West/East African fractions (0.8%) and slightly higher than the combined African fractions (2.1%). South Asian contributions outweigh East Asian contributions (*p* value of < 0.00001, paired Wilcox test) (Additional file 1: Table S2). The South Asian contribution seems to have come predominantly from Indian populations (Additional file 1: Figure S8).

West/East Africans contributed an average of 0.8% of the Afrikaner ancestry (Additional file 1: Table S1) (16.3 out of 2048 ancestors 11 generations ago). Shell [4] estimated that about 63,000 slaves arrived in the Cape colony between 1658 and 1807 and a quarter came from West/East coastal Africa (26.4%, east coast and only 2.5% from West Africa). Only two ships brought West African slaves to the Cape in 1685 [10]. When one takes into account that only 2.5% of African slaves came from West Africa, it is surprising that just over half of this signal is from West Africans rather than East Africans (Additional file 1: Table S2). This discrepancy could possibly be explained by West Africans arriving four generations earlier than East Africans (see Additional file 1: Supplementary Text). More frequent admixture during early years and fast population growth could have caused the genetic footprint of West Africans to exceed that of East Africans. Another explanation that likely contributes to this observation is that a large fraction of the East African slaves brought to the Cape colony might have been East African Bantu speakers and thus they would also trace most of their ancestry to West Africa [54].

The shared allele analysis (Additional file 1: Figure S6) indicates that the West African fraction in the Afrikaners mostly came from West (and possibly East) African slaves and not from southern African Bantu speakers. Afrikaners shared the most alleles with the West African Yoruba from Nigeria, intermediate levels with East African Bantu speakers (LWK from Kenya) and the lowest level with local South African Bantu speakers (southeast Bantu speakers) (Additional file 1: Figure S6). Although current-day South African Bantu speakers trace the majority of their ancestry (80%) to West Africa [35, 39, 54, 55] (Fig. 1), there were no Bantu speakers present in the southwestern part of Africa during colonial times ([13] and Additional file 1: Supplementary Text).

While admixture fractions between East Asians, South Asians and Khoe-San correlate well with each other in Afrikaner individuals (Additional file 1: Figure S1), West/East African fractions do not correlate significantly with South Asian and East Asian fractions and a high number of Afrikaners had no West/East African admixture (26/77). These patterns could possibly be explained by the fact that there were relatively few West African slaves at the Cape, that the arrival of West African slaves was contained in a very limited time period and that East African slaves arrived later in time.

Although the different admixture events into the Afrikaners could not be distinguished in the admixture time estimates (probably because they all occur during the same time period), the estimated time of 9.3 (SD = 0.99) generations (Additional file 1: Table S4) compares well with genealogical estimates. In the Afrikaner population, the average generation time for men is 32.92 years whereas for women it is 27.36 [14]. Using a mean generation time of 30 years, the time of admixture equates to 279 years ago. The average date of birth of the study participants was 1960—which resolves to an estimated admixture date of 1681 (± 30 years). This date falls during early colonial times at the Cape and since most admixing events are thought to have occurred during this time period, the genetic dating falls into the expected range.

Afrikaners showed several clear local genomic signals where allele frequencies significantly deviate from the frequencies of comparative northwestern European groups. A gene associated to the top signal is expressed in the testes and has sperm antigen functions, which might suggest reproduction adaptation in the Afrikaners. Scans for haplotypes under selection in the Afrikaners implicated several genes associated with diet, i.e. intestinal function, lipid and glucose metabolism, possibly indicating adaptation to modified or novel food sources.

It is interesting to observe that Afrikaners do not present a signal of a population bottleneck compared to European groups, even though they had a very small founding population (Fig. 4). This could be explained by the fact that even though the initial founding population of the Afrikaners was small, they were from diverse origins in Europe. Additionally, some of the initial unions resulted in admixed children who were incorporated in the resultant population. The very high population growth rate means that alleles were unlikely to coalesce in the recent past. For example, one Afrikaner individual’s parents (JMG —[30]) had 125 common ancestors, but these were so distant (paths longer than 16 steps) from each other that his inbreeding coefficient is only 0.0019. Until recently, most humans were sedentary and populations were small so that inbreeding due to distant relations was not unusual. However, a number of founder effects for specific diseases have been identified in Afrikaners ([28, 29] and Additional file 1: Supplementary Text). These founder effects however need not imply inbreeding but rather suggest a sampling effect, i.e. some disease alleles were present in original founders and were amplified through exponential population growth.

## Conclusion

Although Afrikaners have the majority of their ancestry from northwestern Europe, non-European admixture signals are ubiquitous in the Afrikaner population. Interesting patterns and similarities could be seen between genealogical predictions and genetic inferences. Afrikaners today have comparable inbreeding levels to current-day European populations. The diverse European origins of the settlers, combined with local Khoe-San admixture and admixture with people that were brought to southern Africa as slaves, might have been some of the factors that helped to counteract the adverse effect of a small founding population size and inbreeding.

## Materials and methods

### Sample collection and genotyping

The 77 individuals included in this study form part of parallel studies on non-paternity [14, 56] and on the mitochondrial DNA heritage of self-identified Afrikaners. Fifty-four samples came from 17 groups of men bearing the same surnames (an average of 3.2 individuals per family with the same surname). Twenty-three samples are from unrelated patrilines, either having unique surnames or stemming from different founders. Males with the same surname will have the same Y-chromosome (sex chromosomes were excluded in the analyses). However, since they are separated by an average of 15.8 generations along their patrilines, we only expect 1.5 × 10^−5^ of their autosomal genomes to be more similar than randomly picked males with different surnames. Furthermore, due to the small group of founders of the Afrikaner population, each male subject included in the study will sample the entire Afrikaner founding population with a high coverage. For instance, one Afrikaner (JMG) is related to 299 founders 1101 times [30]. We can expect any two Afrikaners to be related many times through paths that are in excess of 15 steps [30]. For instance, JMG’s parents have 125 common ancestors, but despite multiple paths running through many of these common ancestors, his inbreeding coefficient is only 0.0019 [30]. Hence, to sample completely unrelated individuals in the Afrikaner population would be impossible, and sampling individuals that are more related via one path (direct patrilines for example) will not affect estimates more than the hundreds of other paths linking random individuals in this recently founded population.

Samples were collected with Oragene® DNA Saliva collection kits (DNA Genotek, Kanata, Canada) and whole genome DNA was extracted according to the manufacturer’s instructions. Final concentrations were adjusted to 50 ng/μl. Genotyping was performed by the SNP&SEQ Technology Platform in Uppsala, Sweden (www.genotyping.se), using the Human Omni 5M SNP array. Results were analysed using the software GenomeStudio 2011.1, and the data were exported to Plink format and aligned to hg19.

### Genotype filtering and merging with comparative data

SNP data quality filtering and merging to comparative data was done with PLINK v1.90b3 [57]. A 10% genotype missingness threshold was applied, and the HWE rejection confidence level was set to 0.001. SNPs with a chromosome position of 0, indels, duplicate-, mitochondrial- and sex chromosome SNPs were removed. All individuals passed a missingness threshold of 15% and a pairwise IBS threshold of 0.25 (for identification of potential relatives).

The resultant dataset of 4,154,029 SNPs and 77 individuals were phased using fastPHASE [58], with 25 haplotype clusters, 25 runs of the EM-algorithm and 10% assumed missingness. Subsequently, the data was merged with the data from Schlebusch et al. [39], containing 2,286,795 quality-filtered autosomal SNPs typed in 117 southern African Khoe-San and Bantu speakers. Before merging the datasets, AT and CG SNPs were removed from the datasets. During the merge, the strands of mismatching SNPs were flipped once and remaining mismatches were removed and only the overlapping positions between the datasets were kept.

To get a more extensive set of African and non-African comparative data, we furthermore downloaded SNP data from the 1000 Genomes Project website, at ftp.1000genomes.ebi.ac.uk/vol1/ftp/technical/working/20120131_omni_genotypes_and_intensities [59]. The 1000 genomes genotype data were quality filtered using the same thresholds as used in our datasets (described above). The following populations were included from the 1000 genomes dataset: YRI and LWK (Yoruba and Luhya—West African ancestry); MKK (Maasai—East African); ACB and ASW (African-Americans in the Caribbeans and southwest USA); TSI, IBS, CEU, GBR and FIN (Tuscans, Iberians, northwest European ancestry, British, Finnish—European); JPT, GIH, CHB, CHS, CDX and KHV (Japanese, Indian ancestry, Han Chinese Beijing, Han Chinese South, Dai Chinese, Vietnamese—Asian); PEL, PUR, MXL and CLM (Peruvians, Puerto Ricans, Mexican ancestry, Colombians—Native American (admixed)). All populations were randomly downsampled to 80 individuals. This merged dataset included a total of 2,182,606 high-quality SNPs in 1747 individuals from 33 populations.

For finer scale resolution of the European component and the Asian component in Afrikaners, this dataset was furthermore merged with (1) the POPRES dataset [48, 49] and (2) various datasets containing populations from east, south and southeast Asia [50, 60–66]. The merged Asian dataset was randomly downsampled to 20 individuals per population. These European and Asian comparative datasets were quality filtered and phased with the same thresholds and parameters as used in the previous datasets. Although these datasets had much lower SNP densities (149,365 SNPs for the European and 313,790 SNPs for the Asian dataset), they contained many more comparative populations (37 European comparative populations for the European dataset and 63 Asian comparative populations for the Asian dataset).

### Population structure analysis

Population genetic analysis was conducted for the main merged dataset, containing 1747 individuals from 33 populations and 2,182,606 SNPs. We inferred admixture fractions [46] in order to investigate genomic relationships amongst individuals based on the SNP genotypes. Default settings and a random seed were used. Between 2 and 10 clusters (*K*) were tested. A total of 100 iterations of ADMIXTURE were run for each value of *K*, and the iterations were analysed using CLUMPP [67] for each *K* to identify common modes amongst replicates. Pairs of replicates yielding a symmetric coefficient *G*’ ≥ 0.9 were considered to belong to common modes. The most frequent common modes were selected and visualized with DISTRUCT [68]. For the Asian, extended dataset similar settings were used as described above; however, clustering was done for *K* = 2 to *K* = 15 due to the higher number of populations in the dataset.

PCA was performed with EIGENSOFT [69] with the following parameters: r2 threshold of 0.1, population size limit of 80 and 10 iterations of outlier removal. Projected PCA analysis was done using EIGENSOFT by constructing principal components (PCs) based on the POPRES dataset [48, 49] and then projecting Afrikaners on existing PCs.

Formal f3 and f4 tests of admixture were done using the ADMIXTOOLS package [47]. We did f3 tests between all pairs of comparative populations specifying (two) potential parental sources of the Afrikaner population. Additionally, we fixed the European source (CEU) to show the best African source and the African source (≠Khomani) to show the best European source. We also did f4 tests to differentiate between Chinese vs. Japanese and West vs. East Africans as best sources. Shared private alleles were inferred using ADZE [70] for all pairwise population combinations of populations with at least 15 individuals.

The date estimations were done using Malder v.1.0, ADMIXTOOLS package v.5.0 [47]. The HapMap II genetic map was used as recombination map.

### Genetic diversity analysis

To estimate genetic diversity and evidence of bottleneck effects in the Afrikaner population, we estimate runs of homozygosity (RoH) across the genome. RoH were calculated using PLINK [57], applying the following parameters: --homozyg --homozyg-window-kb 5000 --homozyg-window-het 1 --homozyg-window-threshold 0.05 --homozyg-kb 500 --homozyg-snp 25 --homozyg-density 50 --homozyg-gap 100.

The above analyses were also repeated on Afrikaner data where the non-European admixed fragments were masked out of Afrikaner genomes. To identify the non-European genomic fragments, we inferred genome local ancestry for the Afrikaner individuals using RFMix version 1.5.4 [71]. The following populations were used as putative sources: CEU, CDX, YRI and Khoe-San groups (combined !Xun, ≠Khomani, Karretjie and Ju|huansi). RFMix was run with the following settings: RFMix_v1.5.4/RunRFMix.py --forward-backward -e 2 infilename. Other settings were left as default.

### Patterns of selection and allele frequency differences

We scanned Afrikaner genomes for genome local patterns of allele frequency variation by doing a locus-specific branch length (LSBL) analysis. LSBL values were calculated for the AFR compared to two European populations (CEU and GBR). The first pairwise Fst were calculated between the three populations in Plink v1.90b4.9. To then arrive at the LSBL value, the Fst between CEU and GBR was added to the Fst between AFR and GBR and the Fst between AFR and CEU was subtracted. This sum is then divided by two.

To scan for signals of genome-wide selection in the Afrikaner group, integrated haplotype scores (iHS) and the cross population extended haplotype homozygosity (XP-EHH) were analysed using the R package REHH [72]. The ancestral state was identified by its presence in the chimpanzee, gorilla, orangutan and human genomes (downloaded from UCSC). Based on this requirement, we performed selection analyses on 1,759,008 SNPs. iHS and XP-EHH were calculated with maximum distance between two SNPs of 200,000 bp. For the XP-EHH, we compared the Afrikaners (AFR) haplotype homozygosity with Northwest European ancestry individuals (CEU).

## Supplementary information

**Additional file 1: Figure S1.** Non-European admixture fractions (of K=6) sorted by ancestry fraction. **Figure S2.** Principal component analysis for PC1-PC10 and the variation explained by PCs. **Figure S3.** Results from f3-test. Populations are colored according to regional affiliation. **Figure S4.** Results from f3-test. The CEU (A) and Khomani (B) populations are fixed to show the best African and non-African sources to the Afrikaner population. **Figure S5.** Fraction of shared private alleles between the Afrikaner population and a comparative population. **Figure S6.** Shared private alleles between the Afrikaner populations and populations with West-African ancestry. **Figure S7.** A) Afrikaner individuals (black circles) projected on a PCA based on European genetic variation from the POPRES dataset. B) Population variation on PC 1 and 2 summarized as averages and standard deviations. **Figure S8.** Admixture analyses of the Asian extended dataset. **Figure S9.** Manhattan plot of Locus specific branch length (LSBL) results. **Figure S10.** Manhattan plots of selection scan results. **Figure S11.** Runs of of Homozygosity (RoH) for European populations and the Afrikaner population. **Table S1.** Admixture fractions of the Afrikaner individuals at K=6 (ADMIXTURE). **Table S2.** Admixture fractions of the Afrikaner individuals at K=9 (ADMIXTURE). **Table S3.** f4 test to test specific sources of ancestry in the Afrikaner population. **Table S4.** Admixture LD decay estimate of admixture times into the Afrikaner population done in Malder. **Table S5.** Top 5 peaks detected with Locus Specific Branch Length scans (indicating allele frequency differences of AFR compared to CEU and GBR). **Table S6.** Top 5 selection scan peaks detected with iHS and XP-EHH scans. **Supplementary Text.** This supplementary note discusses the populations from which the Afrikaner population arose, it summarizes genealogical information of admixture and presents genetic information of admixture. **Figure S12.** The population size of adult Afrikaner men (solid heavy line) and women (solid lighter line) as a function of time. **Figure S13.** The origins of slaves arriving in the Cape. **Table S7.** Four estimates from three studies of the percentage composition of Afrikaners.

## Data Availability

All data generated or analysed during this study are included in this published article, its supplementary information files and publicly available repositories. The anonymized genome-wide SNP data of 75 of the 77 Afrikaner individuals who consented to have their data shared electronically will be made available for research use through the ArrayExpress database (https://www.ebi.ac.uk/arrayexpress) [73], access number E-MTAB-8757. The genetic data of the remaining two individuals will be made available on request after the completion of a Data Access agreement.
