# Supplementary material for: Patterns of African and Asian admixture in the Afrikaner population of South Africa
Source: BMC Biol. 2020 Feb 24;18:16. doi: 10.1186/s12915-020-0746-1 (PMC7038537; doi:10.1186/s12915-020-0746-1)
Supplement: Supplementary file 1 — Additional file 1: Figure S1. Non-European admixture fractions (of K=6) sorted by ancestry fraction. Figure S2. Principal component analysis for PC1-PC10 and the variation explained by PCs. Figure S3. Results from f3-test. Populations are colored according to regional affiliation. Figure S4. Results from f3-test. The CEU (A) and Khomani (B) populations are fixed to show the best African and non-African sources to the Afrikaner population. Figure S5. Fraction of shared private alleles between the Afrikaner population and a comparative population. Figure S6. Shared private alleles between the Afrikaner populations and populations with West-African ancestry. Figure S7. A) Afrikaner individuals (black circles) projected on a PCA based on European genetic variation from the POPRES dataset. B) Population variation on PC 1 and 2 summarized as averages and standard deviations. Figure S8. Admixture analyses of the Asian extended dataset. Figure S9. Manhattan plot of Locus specific branch length (LSBL) results. Figure S10. Manhattan plots of selection scan results. Figure S11. Runs of of Homozygosity (RoH) for European populations and the Afrikaner population. Table S1. Admixture fractions of the Afrikaner individuals at K=6 (ADMIXTURE). Table S2. Admixture fractions of the Afrikaner individuals at K=9 (ADMIXTURE). Table S3. f4 test to test specific sources of ancestry in the Afrikaner population. Table S4. Admixture LD decay estimate of admixture times into the Afrikaner population done in Malder. Table S5. Top 5 peaks detected with Locus Specific Branch Length scans (indicating allele frequency differences of AFR compared to CEU and GBR). Table S6. Top 5 selection scan peaks detected with iHS and XP-EHH scans. Supplementary Text. This supplementary note discusses the populations from which the Afrikaner population arose, it summarizes genealogical information of admixture and presents genetic information of admixture. Figure S12. The population size of adult Afrikaner men ( [file 12915_2020_746_MOESM1_ESM.pdf]

SUPPLEMENTARY MATERIAL

Patterns of African and Asian admixture in the Afrikaner population of South Africa

N Hollfelder[1*], JC Erasmus[2*], R Hammaren[1], M Vicente[1], M Jakobsson[1,3,4+], JM Greeff[2#+], CM Schlebusch[1,3,4#+]

[1] Human Evolution, Department of Organismal Biology, Uppsala University, Norbyvägen 18C, SE-752 36 Uppsala, Sweden

[2] Department of Biochemistry, Genetics and Microbiology, University of Pretoria, Pretoria, 0002, South Africa

[3] Science for Life Laboratory, Uppsala University, Norbyvägen 18C, SE-752 36 Uppsala, Sweden

[4] Palaeo-Research Institute, University of Johannesburg, P.O. Box 524, Auckland Park 2006, South Africa

Running Title: Admixture into the Afrikaner population of South Africa

* Equal contribution
[+] Equal contribution

[#] Correspondence: Carina Schlebusch
Email: carina.schlebusch@ebc.uu.se

[#] Correspondence: Jaco Greeff
Email: jaco.greeff@up.ac.za

Keywords: Afrikaner, South Africa, admixture, slave trade, colonial times

SUPPLEMENTARY FIGURES AND TABLES

Contents

Supplemental Figures ……….pp. 3 - 13
Supplemental Tables ………..pp. 14 – 23
Supplemental Text …………pp. 24 - 30

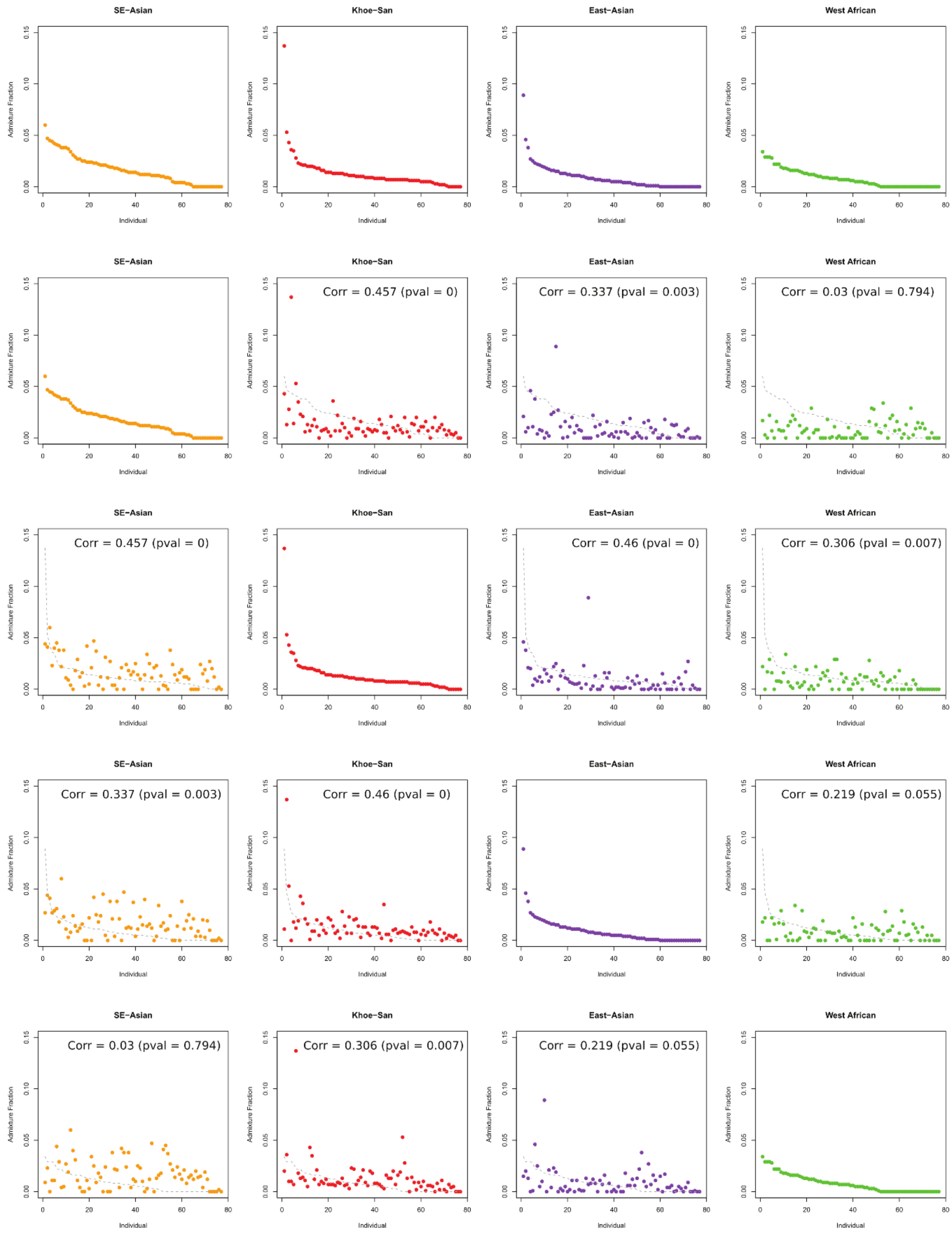

Figure S1: Non-European admixture fractions (of K=6) sorted by ancestry fraction. The first row shows all admixture fractions sorted by their own ancestry, the next row is sorted according to South Asian ancestry, then Khoe-San ancestry, East Asian ancestry, and finally West African ancestry. Ancestry correlations and significance of correlation are indicated on graphs.

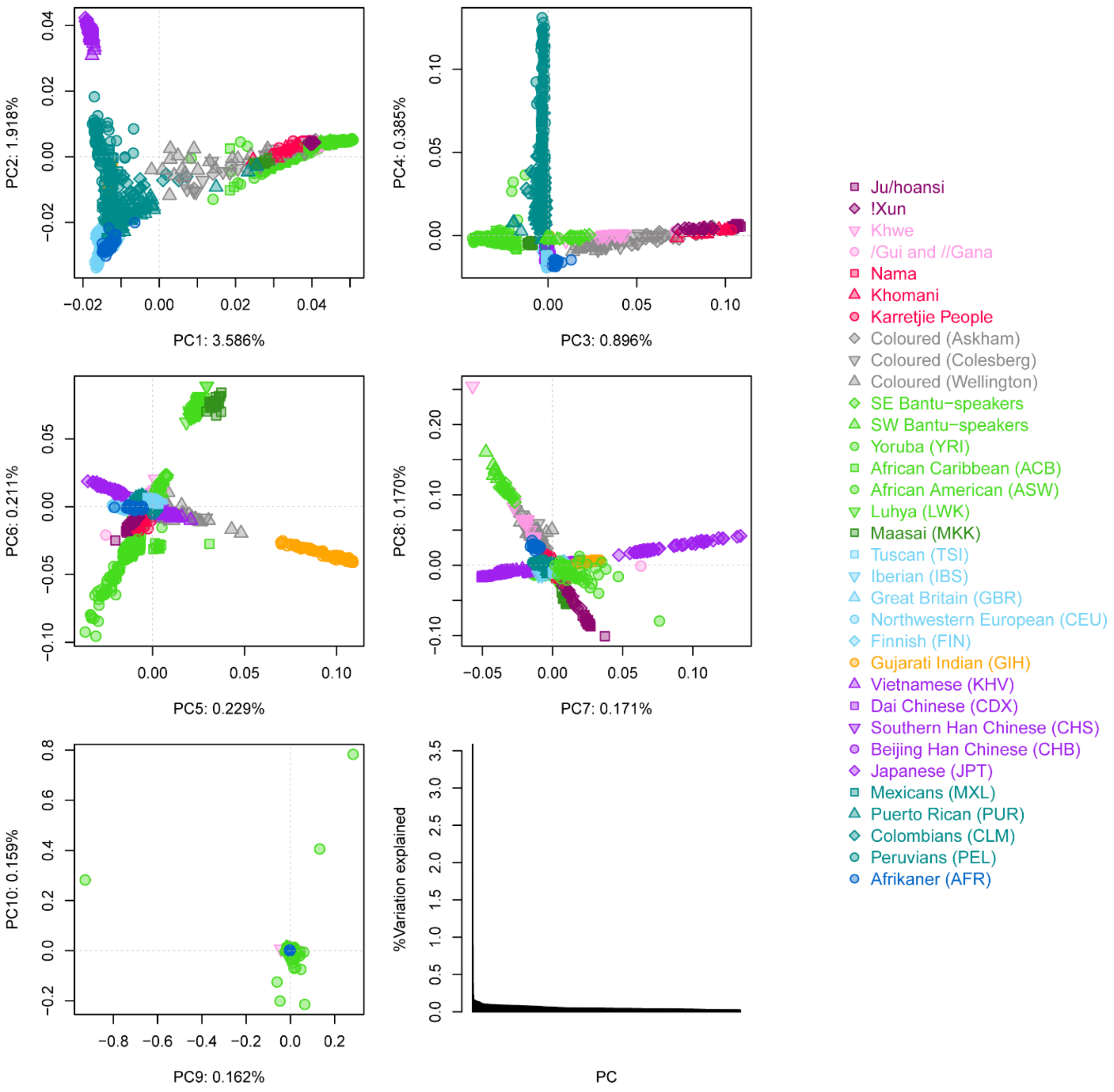

Figure S2: Principal component analysis for PC1-PC10 and the variation explained by PCs. Populations are colored according to regional grouping.

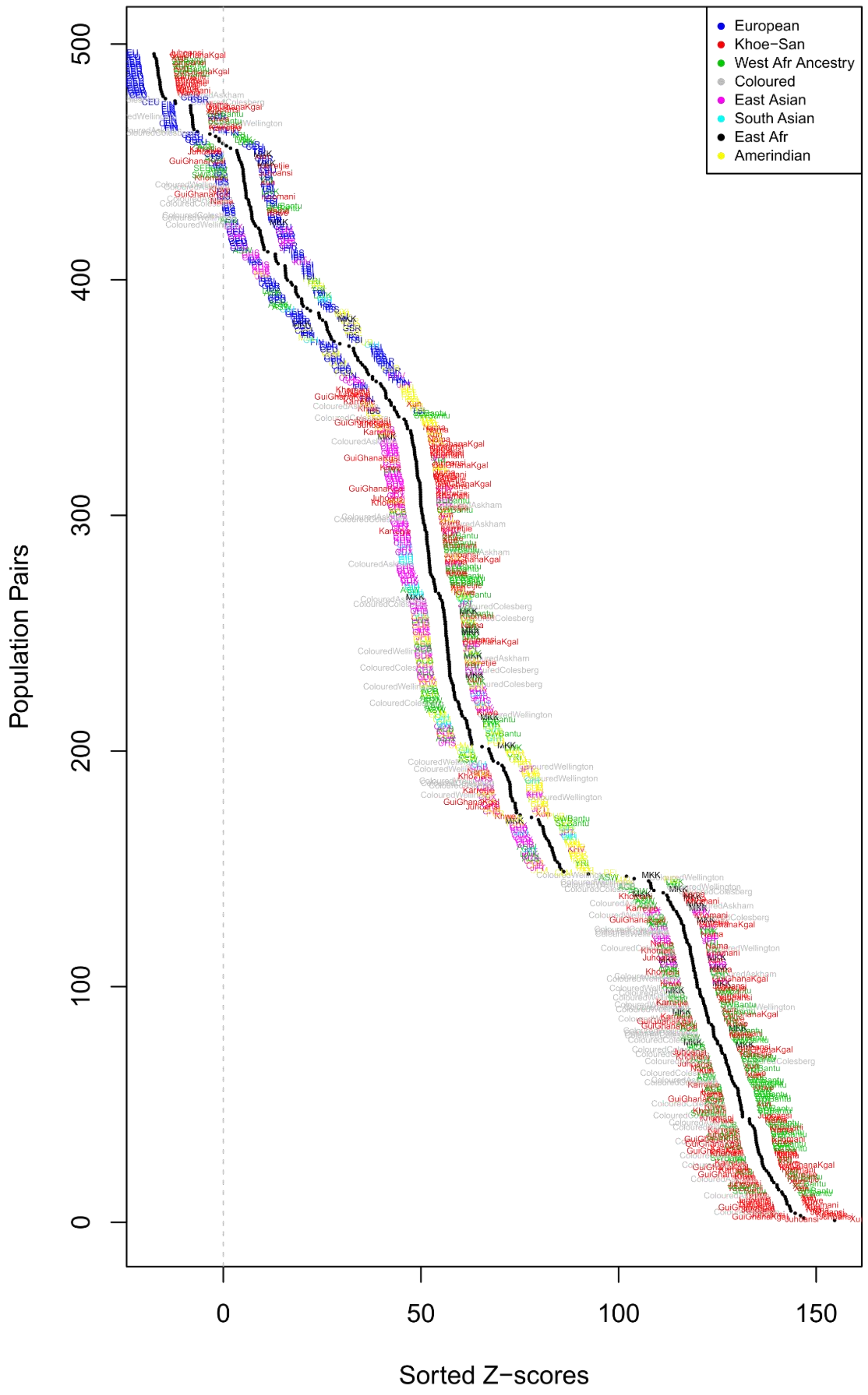

Figure S3: Results from f_3-test. Populations are colored according to regional affiliation.

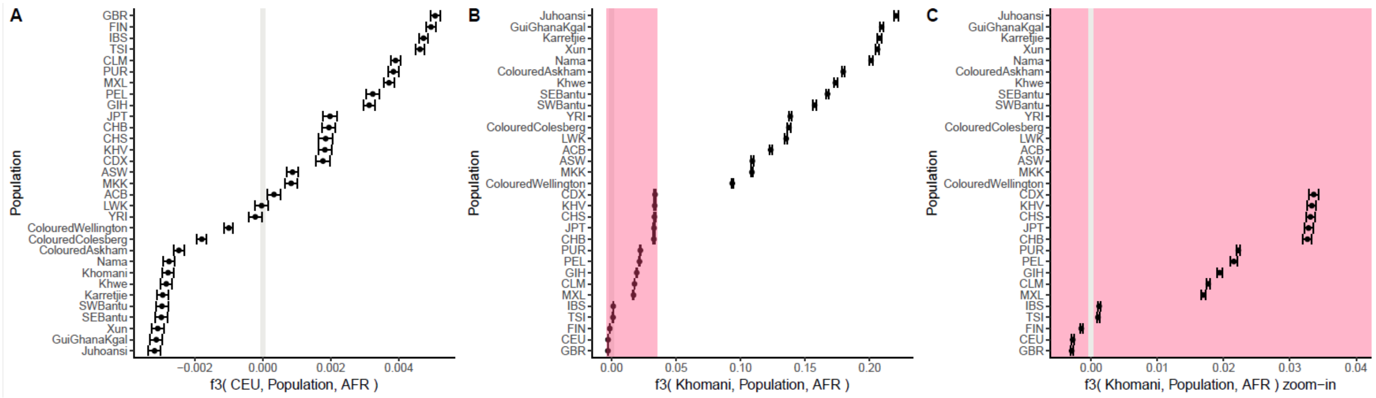

Figure S4: Results from f_3-test. The CEU (A) and Khomani (B) populations are fixed to show the best African and non-African sources to the Afrikaner population. C is a zoom-in of B.

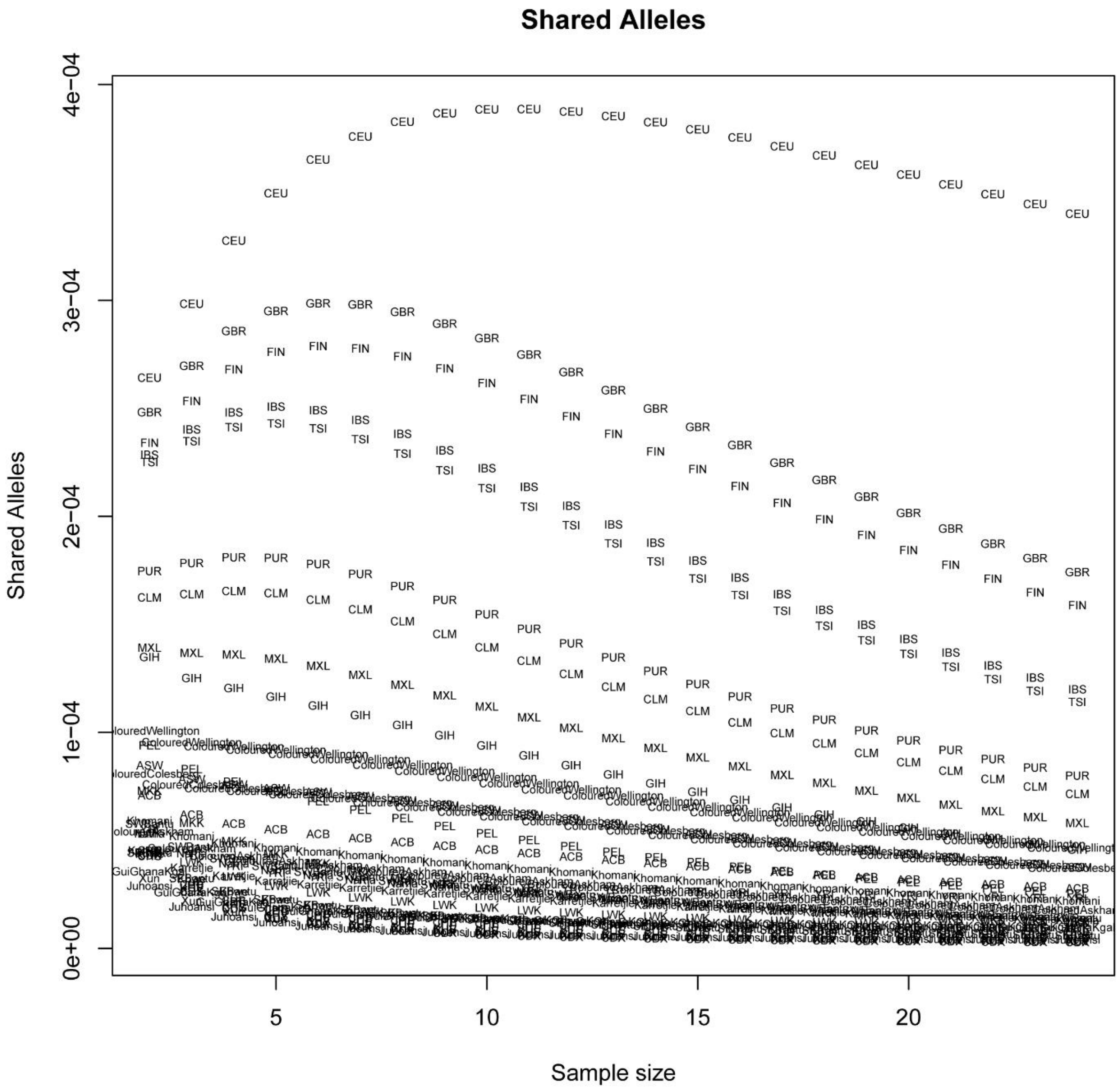

Figure S5: Fraction of shared private alleles between the Afrikaner population and a comparative population.

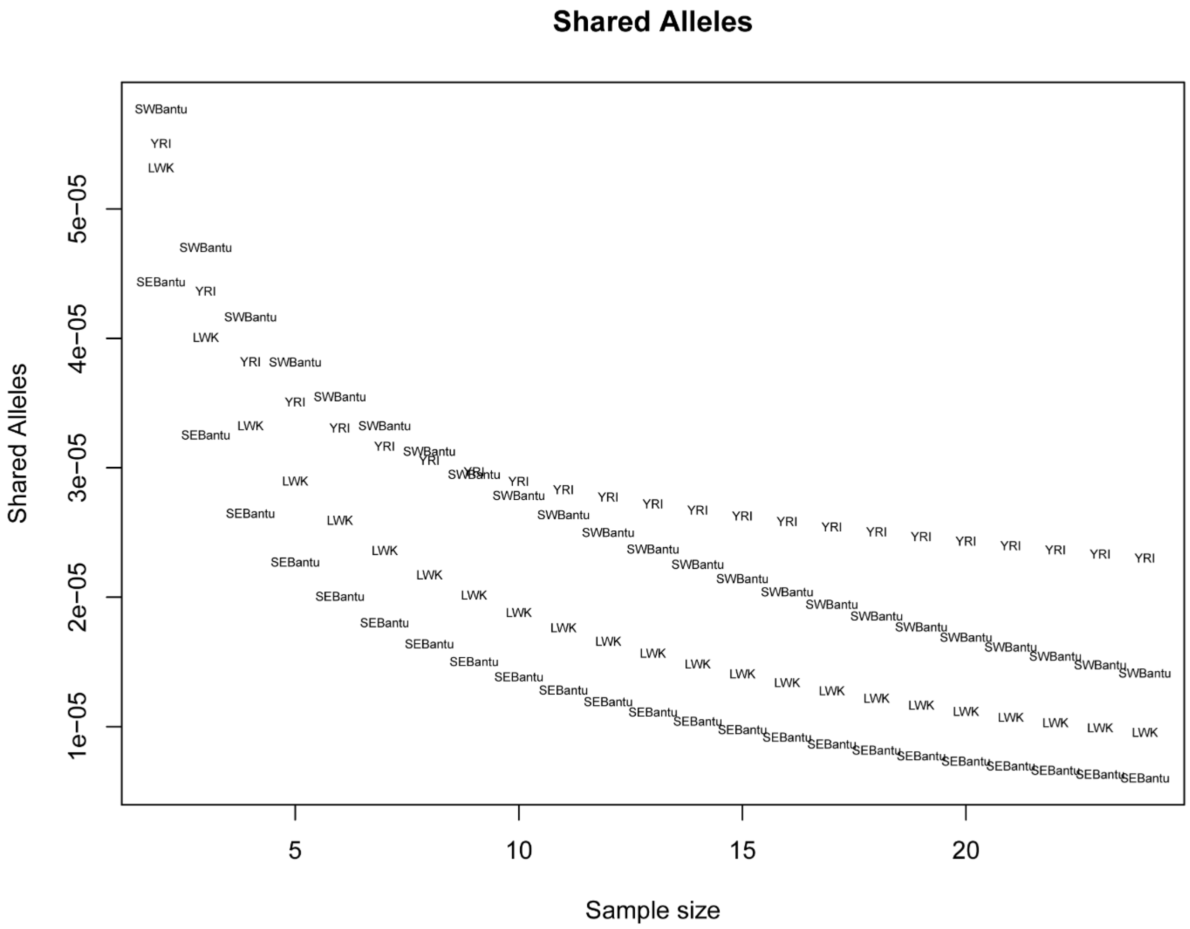

Figure S6: Shared private alleles between the Afrikaner populations and populations with West-African ancestry.

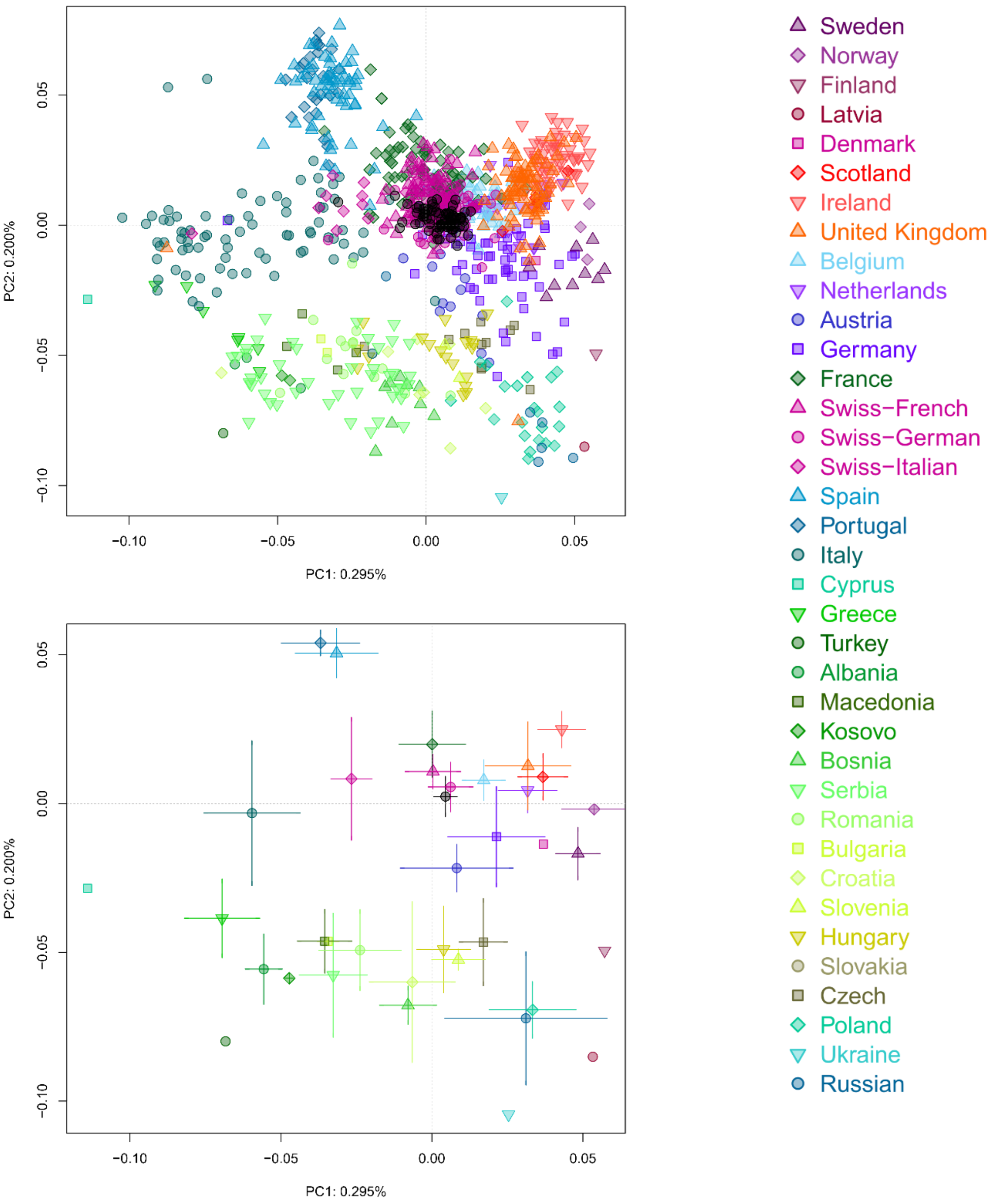

Figure S7: A) Afrikaner individuals (black circles) projected on a PCA based on European genetic variation from the POPRES dataset. B) Population variation on PC 1 and 2 summarized as averages and standard deviations.

A B

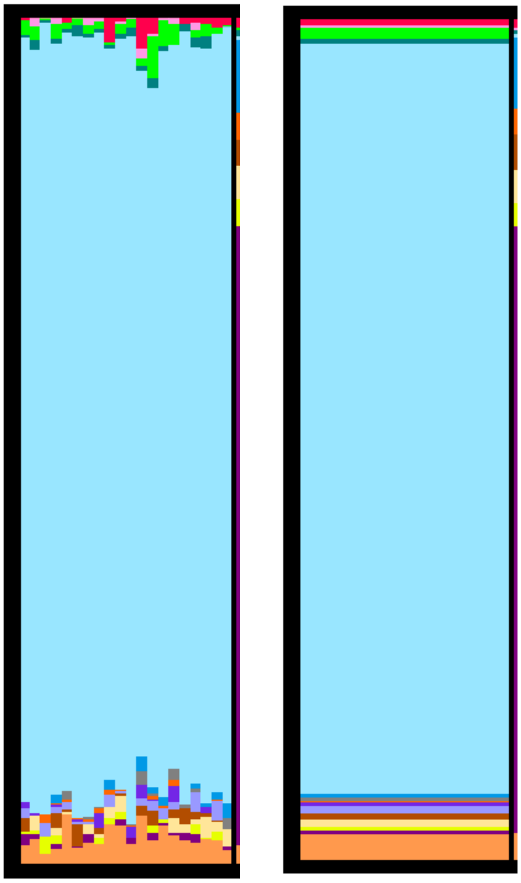

C D

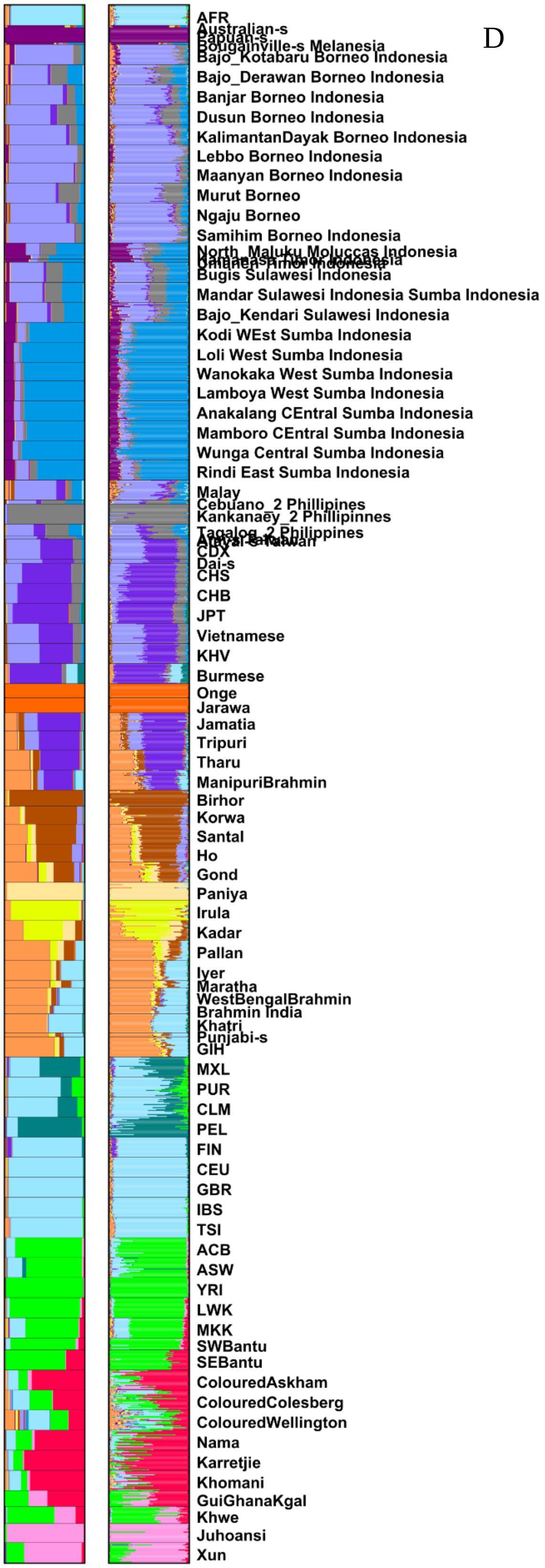

E

Cluster Order	Color	Major cluster in:
1	red	Southern San
2	pink	Northern San
3	light green	West African
4	sea green	Native American
**5**	**light blue**	**European**
6	blue	Sumba Indonesia
7	gray	Phillipines
8	dark orange	Onge (Andaman)
9	purple	Japanese (East Asian)
10	light purple	Borneo Indonesia, Malaysia
11	brown	India (Central, Austro_asiatic)
12	light yellow	India (South, Dravidian)
13	yellow	India (South, Dravidian)
14	maroon	Australian and Papuan
15	orange	Indian (Upper Caste, Indo-European speaking)

Figure S8: Admixture analyses of the Asian extended dataset. Clustering at K=15. Panels A and B show a zoom-in of the AFR individuals, with A the individual clustering and B the population average clustering. Panels C and D show clustering at K=15 for the entire Asian extended dataset, with population average clustering (C) and individual clustering (D). Clusters were sorted so that non-Asian admixture are shown above the European component (light blue component) and Asian admixture below the European component at the bottom of the panel, according to the order shown in E.

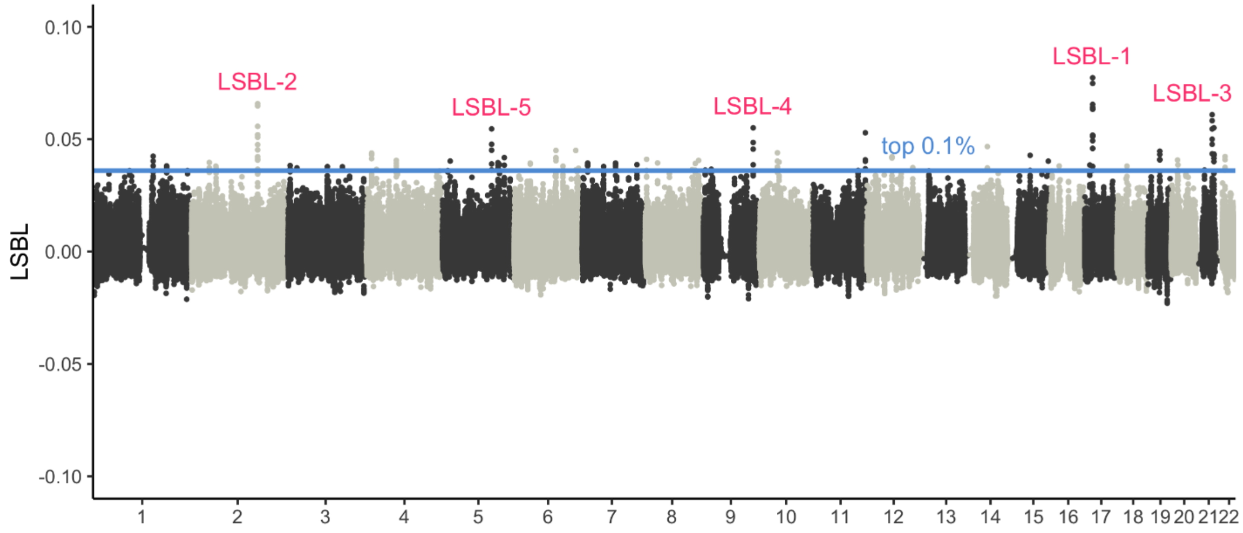

Figure S9: Manhattan plot of Locus specific branch length (LSBL) results. LSBL were calculated between AFR and comparative groups, CEU and GBR. The X-axis indicate chromosomes and genomic positions and the Y-axis indicate the LSBL value (Materials and Methods). The top five peaks are labelled and defined more in Table S5.

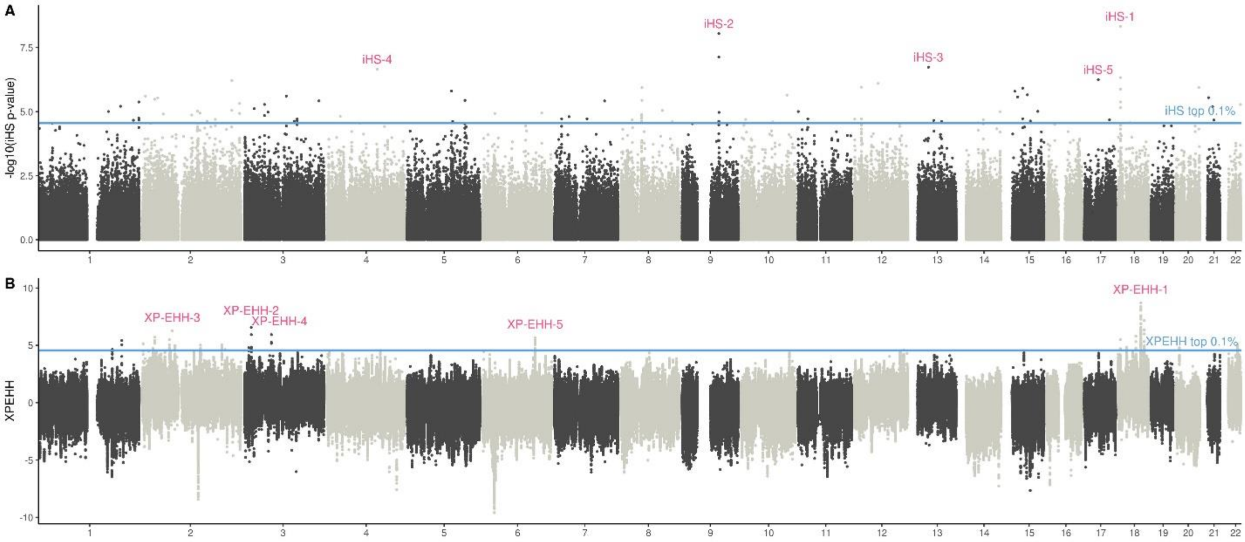

Figure S10: Manhattan plots of selection scan results. A, iHS results and B, XP-EHH results. XP-EHH were calculated between AFR and CEU. Regions of extended homozygosity of AFR (compared to CEU) are indicated above the line and regions of extended homozygosity in CEU (compared to AFR) are indicated below the line. The X-axis indicate chromosomes and genomic positions and the Y-axis indicate –log10(p-value). The top five peaks are labelled and defined more in Table S6.

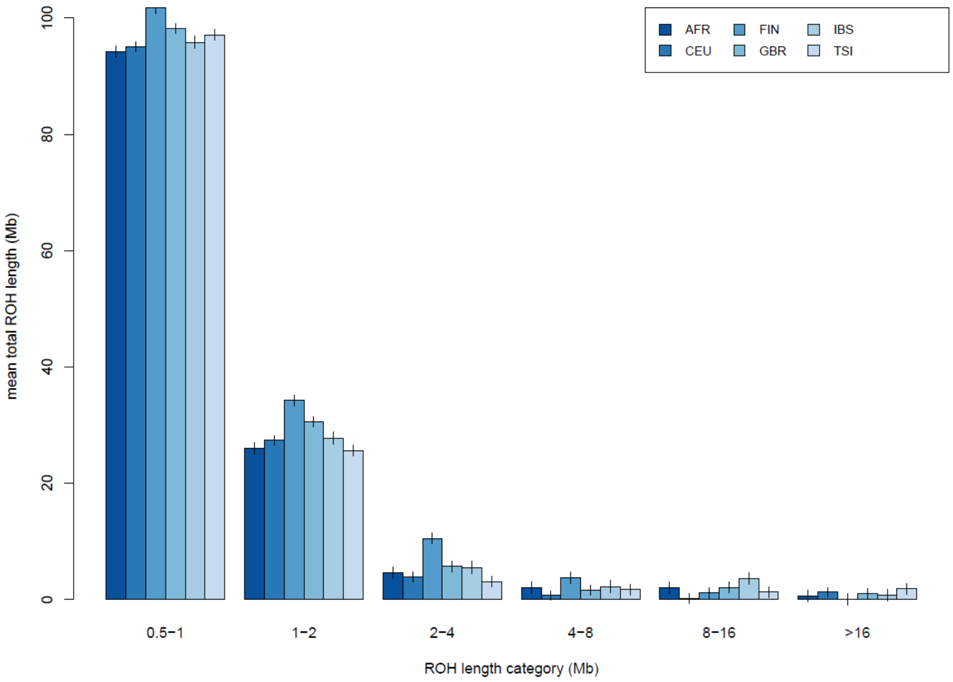

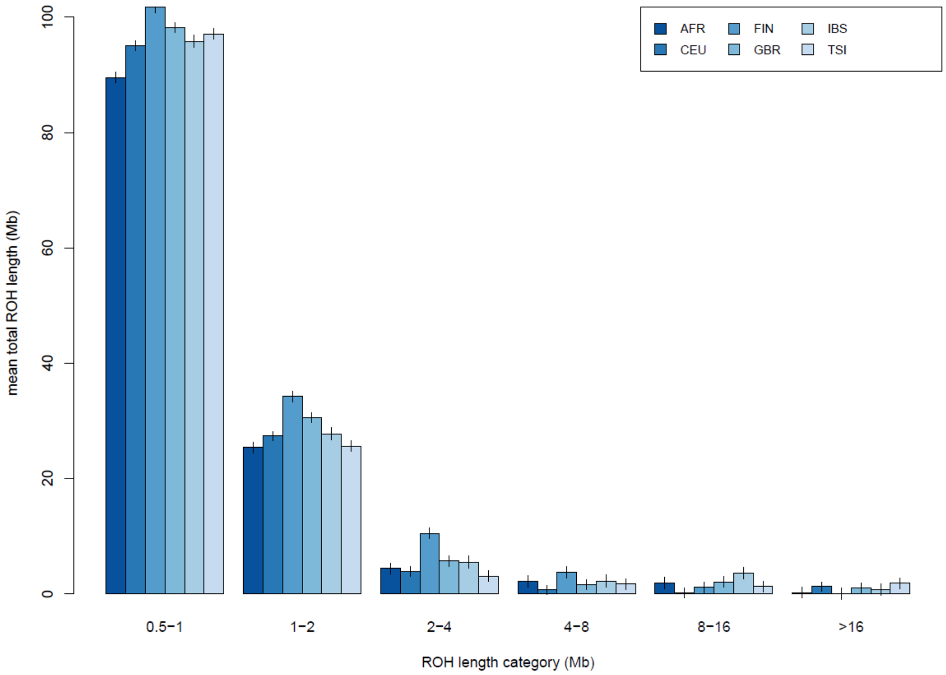

Figure S11: Runs of of Homozygosity (RoH) for European populations and the Afrikaner population. The top plot contains the RoH without any ancestry masking and in the bottom plot all non-European parts of Afrikaner genomes were masked-out before running RoH.

Table S1: Admixture fractions of the Afrikaner individuals at K=6 (ADMIXTURE). Sorted by total fraction of Non-European ancestry.

ID	European	Total other Admixture	South-Asian	Khoe-San	East Asian	West/East African	Native American
122	0.751	0.249	0.044	0.137	0.046	0.022	0.000
7	0.851	0.149	0.060	0.043	0.021	0.017	0.008
247	0.854	0.146	0.027	0.011	0.089	0.018	0.000
87	0.868	0.132	0.041	0.053	0.038	0.000	0.000
109	0.891	0.109	0.023	0.036	0.020	0.029	0.000
162	0.902	0.098	0.029	0.018	0.025	0.022	0.004
263	0.905	0.095	0.040	0.035	0.004	0.016	0.000
28	0.916	0.084	0.009	0.020	0.015	0.034	0.007
67	0.917	0.083	0.045	0.028	0.010	0.000	0.000
199	0.918	0.082	0.031	0.012	0.023	0.016	0.000
192	0.919	0.081	0.038	0.023	0.008	0.008	0.005
259	0.924	0.076	0.042	0.014	0.011	0.007	0.003
203	0.927	0.073	0.038	0.021	0.007	0.007	0.000
64	0.930	0.070	0.047	0.013	0.006	0.003	0.001
256	0.933	0.067	0.011	0.021	0.019	0.016	0.000
258	0.936	0.064	0.022	0.022	0.012	0.008	0.000
234	0.940	0.060	0.018	0.019	0.022	0.001	0.001
255	0.944	0.056	0.024	0.009	0.016	0.007	0.000
128	0.945	0.056	0.034	0.007	0.002	0.012	0.000
601	0.945	0.055	0.025	0.007	0.011	0.012	0.000
14	0.945	0.055	0.037	0.013	0.005	0.000	0.000
2	0.945	0.055	0.016	0.016	0.013	0.002	0.007
76	0.946	0.054	0.027	0.000	0.027	0.000	0.000
200	0.948	0.052	0.005	0.014	0.010	0.019	0.005
111	0.949	0.052	0.000	0.010	0.013	0.029	0.000
81	0.949	0.051	0.038	0.006	0.000	0.007	0.000
155	0.951	0.049	0.011	0.010	0.000	0.029	0.000
215	0.952	0.048	0.018	0.009	0.011	0.011	0.000
51	0.952	0.048	0.011	0.007	0.001	0.028	0.002
238	0.954	0.046	0.019	0.005	0.000	0.018	0.004
241	0.956	0.044	0.004	0.013	0.005	0.022	0.000
46	0.957	0.043	0.021	0.007	0.008	0.008	0.000
261	0.957	0.043	0.021	0.014	0.007	0.000	0.000
605	0.958	0.042	0.011	0.012	0.005	0.013	0.001
604	0.959	0.041	0.003	0.016	0.018	0.004	0.000
154	0.961	0.040	0.014	0.009	0.016	0.000	0.000
260	0.961	0.039	0.025	0.008	0.000	0.005	0.001
229	0.961	0.039	0.024	0.006	0.001	0.009	0.000
22	0.961	0.039	0.012	0.018	0.000	0.006	0.002
49	0.961	0.039	0.023	0.007	0.005	0.005	0.000
89	0.962	0.038	0.004	0.020	0.008	0.007	0.000

120	0.963	0.037	0.024	0.002	0.011	0.000	0.000
262	0.964	0.036	0.000	0.020	0.012	0.003	0.001
1	0.966	0.034	0.012	0.013	0.006	0.004	0.000
194	0.967	0.033	0.014	0.008	0.002	0.010	0.000
218	0.967	0.033	0.017	0.009	0.002	0.000	0.005
21	0.967	0.033	0.012	0.005	0.015	0.000	0.001
35	0.969	0.031	0.021	0.010	0.000	0.000	0.000
144	0.970	0.030	0.000	0.007	0.013	0.009	0.000
264	0.970	0.030	0.013	0.007	0.006	0.003	0.000
129	0.971	0.029	0.010	0.008	0.002	0.006	0.004
138	0.972	0.028	0.000	0.010	0.003	0.015	0.000
97	0.973	0.027	0.008	0.001	0.017	0.000	0.002
153	0.973	0.027	0.014	0.006	0.004	0.000	0.003
225	0.973	0.027	0.004	0.007	0.000	0.016	0.000
160	0.974	0.026	0.009	0.006	0.000	0.012	0.000
18	0.974	0.026	0.016	0.006	0.004	0.000	0.000
24	0.976	0.025	0.000	0.013	0.001	0.010	0.000
201	0.977	0.023	0.000	0.008	0.001	0.014	0.000
267	0.977	0.023	0.012	0.005	0.006	0.000	0.000
254	0.977	0.023	0.004	0.011	0.000	0.008	0.000
19	0.977	0.023	0.020	0.000	0.000	0.000	0.003
189	0.978	0.023	0.003	0.007	0.000	0.013	0.000
226	0.978	0.022	0.014	0.008	0.001	0.000	0.000
178	0.978	0.022	0.015	0.002	0.005	0.000	0.000
56	0.978	0.022	0.004	0.011	0.003	0.000	0.004
219	0.979	0.021	0.019	0.002	0.000	0.000	0.000
180	0.980	0.020	0.010	0.005	0.000	0.005	0.000
94	0.981	0.020	0.000	0.003	0.007	0.009	0.000
57	0.983	0.017	0.012	0.000	0.004	0.000	0.000
150	0.984	0.017	0.000	0.007	0.009	0.000	0.001
265	0.990	0.010	0.000	0.004	0.000	0.000	0.005
137	0.991	0.009	0.000	0.003	0.000	0.005	0.000
257	0.995	0.005	0.000	0.005	0.000	0.000	0.000
86	0.997	0.003	0.000	0.000	0.001	0.000	0.002
53	0.997	0.003	0.002	0.000	0.000	0.000	0.001
31	1.000	0.000	0.000	0.000	0.000	0.000	0.000
**Mean**	**0.953**	**0.047**	**0.017**	**0.013**	**0.009**	**0.008**	**0.001**
**SD**	**0.038**	**0.038**	**0.014**	**0.017**	**0.013**	**0.009**	**0.002**
**Median**	**0.961**	**0.039**	**0.014**	**0.009**	**0.005**	**0.006**	**0.000**
**MIN**	**0.751**	**0.000**	**0.000**	**0.000**	**0.000**	**0.000**	**0.000**
**MAX**	**1.000**	**0.249**	**0.060**	**0.137**	**0.089**	**0.034**	**0.008**
**Count if 0**	**0**	**1**	**12**	**6**	**17**	**26**	**51**

Table S2: Admixture fractions of the Afrikaner individuals at K=9 (ADMIXTURE). Sorted by total fraction of non-European ancestry.

ID	North Eur	South Eur	Total other Admixture	SE Asian	Khoe-San	West Afr	Chinese	East Afr	Japanese	Native American
122	0.403	0.354	0.243	0.040	0.133	0.000	0.039	0.031	0.000	0.000
7	0.466	0.396	0.139	0.061	0.040	0.005	0.016	0.017	0.000	0.000
247	0.482	0.381	0.137	0.029	0.009	0.018	0.080	0.000	0.000	0.000
87	0.493	0.384	0.123	0.042	0.053	0.000	0.028	0.000	0.000	0.000
109	0.439	0.461	0.100	0.024	0.034	0.022	0.010	0.011	0.000	0.000
162	0.427	0.484	0.089	0.030	0.016	0.021	0.020	0.001	0.000	0.000
263	0.447	0.468	0.085	0.036	0.034	0.015	0.000	0.000	0.000	0.000
67	0.467	0.458	0.075	0.046	0.027	0.000	0.002	0.000	0.000	0.000
28	0.443	0.483	0.074	0.010	0.017	0.028	0.009	0.009	0.000	0.001
199	0.442	0.485	0.073	0.031	0.010	0.016	0.016	0.000	0.000	0.000
192	0.469	0.460	0.071	0.040	0.021	0.000	0.000	0.009	0.000	0.000
259	0.490	0.444	0.067	0.042	0.013	0.000	0.004	0.008	0.000	0.000
203	0.464	0.472	0.064	0.038	0.020	0.006	0.000	0.000	0.000	0.000
64	0.495	0.444	0.061	0.046	0.010	0.000	0.000	0.005	0.000	0.000
256	0.509	0.433	0.058	0.011	0.018	0.001	0.010	0.020	0.000	0.000
258	0.459	0.485	0.056	0.022	0.020	0.000	0.005	0.009	0.000	0.000
234	0.484	0.464	0.052	0.019	0.017	0.000	0.013	0.003	0.000	0.000
128	0.411	0.541	0.048	0.028	0.003	0.000	0.000	0.016	0.000	0.000
255	0.488	0.464	0.048	0.024	0.008	0.006	0.009	0.000	0.000	0.000
76	0.485	0.468	0.047	0.030	0.000	0.000	0.011	0.000	0.006	0.000
601	0.482	0.471	0.047	0.027	0.005	0.011	0.003	0.000	0.000	0.000
14	0.490	0.464	0.045	0.034	0.011	0.000	0.000	0.000	0.000	0.000
2	0.513	0.444	0.043	0.020	0.016	0.000	0.008	0.000	0.000	0.000
111	0.484	0.473	0.043	0.000	0.006	0.019	0.004	0.014	0.000	0.000
81	0.506	0.452	0.042	0.032	0.004	0.005	0.000	0.001	0.000	0.000
200	0.504	0.455	0.041	0.007	0.012	0.019	0.003	0.000	0.000	0.000
215	0.499	0.461	0.040	0.019	0.007	0.003	0.002	0.010	0.000	0.000
155	0.493	0.468	0.039	0.003	0.008	0.028	0.000	0.000	0.000	0.000
51	0.499	0.464	0.038	0.005	0.005	0.028	0.000	0.000	0.000	0.000
46	0.491	0.473	0.036	0.022	0.005	0.000	0.000	0.009	0.000	0.000
238	0.472	0.493	0.036	0.015	0.003	0.018	0.000	0.000	0.000	0.000
241	0.485	0.480	0.035	0.001	0.010	0.016	0.000	0.009	0.000	0.000
261	0.441	0.524	0.034	0.021	0.013	0.000	0.001	0.000	0.000	0.000
605	0.453	0.513	0.034	0.009	0.010	0.006	0.000	0.008	0.000	0.000
604	0.509	0.460	0.032	0.005	0.015	0.003	0.010	0.000	0.000	0.000
229	0.531	0.437	0.031	0.017	0.003	0.000	0.000	0.012	0.000	0.000
154	0.476	0.494	0.031	0.012	0.007	0.000	0.010	0.001	0.000	0.000
49	0.469	0.500	0.031	0.021	0.005	0.000	0.000	0.005	0.000	0.000
260	0.521	0.450	0.030	0.018	0.007	0.000	0.000	0.004	0.000	0.000
120	0.471	0.500	0.029	0.024	0.000	0.000	0.005	0.000	0.000	0.000
89	0.521	0.450	0.029	0.002	0.017	0.000	0.000	0.010	0.000	0.000
22	0.490	0.481	0.028	0.004	0.016	0.000	0.000	0.008	0.000	0.000

262	0.492	0.482	0.027	0.000	0.018	0.000	0.005	0.003	0.000	0.000
194	0.514	0.461	0.025	0.010	0.006	0.007	0.000	0.003	0.000	0.000
1	0.479	0.496	0.025	0.009	0.011	0.000	0.000	0.005	0.000	0.000
21	0.555	0.422	0.024	0.014	0.003	0.000	0.007	0.000	0.000	0.000
138	0.450	0.528	0.023	0.000	0.009	0.013	0.000	0.001	0.000	0.000
218	0.472	0.505	0.023	0.015	0.008	0.000	0.000	0.000	0.000	0.000
35	0.515	0.463	0.022	0.013	0.009	0.000	0.000	0.000	0.000	0.000
24	0.537	0.442	0.021	0.000	0.011	0.004	0.000	0.006	0.000	0.000
264	0.469	0.511	0.021	0.013	0.006	0.002	0.000	0.000	0.000	0.000
225	0.515	0.465	0.020	0.000	0.005	0.015	0.000	0.000	0.000	0.000
201	0.467	0.513	0.020	0.000	0.007	0.012	0.000	0.001	0.000	0.000
144	0.496	0.484	0.020	0.001	0.007	0.005	0.004	0.003	0.000	0.000
129	0.509	0.472	0.020	0.006	0.005	0.000	0.000	0.009	0.000	0.000
189	0.512	0.469	0.020	0.000	0.003	0.000	0.000	0.016	0.000	0.000
18	0.490	0.492	0.018	0.014	0.004	0.000	0.000	0.000	0.000	0.000
153	0.448	0.534	0.018	0.013	0.005	0.000	0.000	0.000	0.000	0.000
160	0.502	0.481	0.018	0.000	0.003	0.002	0.000	0.013	0.000	0.000
97	0.500	0.483	0.017	0.008	0.000	0.000	0.010	0.000	0.000	0.000
254	0.502	0.482	0.016	0.000	0.010	0.006	0.000	0.000	0.000	0.000
178	0.500	0.487	0.014	0.014	0.000	0.000	0.000	0.000	0.000	0.000
19	0.464	0.522	0.013	0.013	0.000	0.000	0.000	0.000	0.000	0.000
267	0.501	0.486	0.013	0.011	0.003	0.000	0.000	0.000	0.000	0.000
226	0.474	0.514	0.013	0.007	0.005	0.000	0.000	0.000	0.000	0.000
56	0.479	0.509	0.012	0.001	0.010	0.000	0.000	0.000	0.000	0.001
219	0.472	0.517	0.012	0.012	0.000	0.000	0.000	0.000	0.000	0.000
180	0.498	0.492	0.010	0.001	0.002	0.000	0.000	0.007	0.000	0.000
94	0.474	0.517	0.009	0.000	0.003	0.006	0.001	0.000	0.000	0.000
57	0.445	0.547	0.008	0.008	0.000	0.000	0.000	0.000	0.000	0.000
150	0.500	0.492	0.008	0.002	0.006	0.000	0.000	0.000	0.000	0.000
137	0.514	0.481	0.005	0.000	0.003	0.000	0.000	0.002	0.000	0.000
265	0.450	0.548	0.002	0.000	0.002	0.000	0.000	0.000	0.000	0.000
257	0.519	0.479	0.002	0.000	0.002	0.000	0.000	0.000	0.000	0.000
31	0.479	0.521	0.000	0.000	0.000	0.000	0.000	0.000	0.000	0.000
53	0.497	0.503	0.000	0.000	0.000	0.000	0.000	0.000	0.000	0.000
86	0.490	0.510	0.000	0.000	0.000	0.000	0.000	0.000	0.000	0.000
**Mean**	**0.483599**	**0.477206**	**0.039162**	**0.014923**	**0.011065**	**0.004751**	**0.004471**	**0.003847**	**0.000083**	**0.000022**
**SD**	**0.027902**	**0.035821**	**0.037591**	**0.014403**	**0.017047**	**0.007833**	**0.011060**	**0.005963**	**0.000729**	**0.000142**
**Median**	**0.488300**	**0.480700**	**0.029500**	**0.012000**	**0.006800**	**0.000000**	**0.000000**	**0.000000**	**0.000000**	**0.000000**
**MIN**	**0.403100**	**0.354400**	**0.000000**	**0.000000**	**0.000000**	**0.000000**	**0.000000**	**0.000000**	**0.000000**	**0.000000**
**MAX**	**0.554600**	**0.548400**	**0.242600**	**0.060500**	**0.133200**	**0.028400**	**0.080200**	**0.030800**	**0.006400**	**0.001100**
**Count if 0**	**0**	**0**	**3**	**16**	**9**	**46**	**47**	**40**	**76**	**75**

Table S3: f4 test to test specific sources of ancestry in the Afrikaner population

PopW	PopX	PopY	PopZ	f4	SE	Z-Score
CDX	JPT	AFR	Chimp	-0.000149	4.58E-05	-3.254577
CHB	JPT	AFR	Chimp	5.95E-05	3.50E-05	1.6994485
YRI	MKK	AFR	Chimp	-0.007041	8.90E-05	-79.07498
YRI	LWK	AFR	Chimp	-0.000969	3.98E-05	-24.37351

Table S4: Admixture LD decay estimate of admixture times into the Afrikaner population done in Malder. Values sorted by amplitude and the top 30 values are shown

Reference populations	Amplitude	SD	Z-score	Time	SD	Z-score
GBR;Juhoansi	0.00005317	3.54E-06	15.0045	9.29548	0.987752	9.41074
CEU;Juhoansi	0.00005312	3.64E-06	14.5945	9.29548	0.987752	9.41074
FIN;Juhoansi	0.00005226	3.34E-06	15.6412	9.29548	0.987752	9.41074
GBR;Xun	0.00005068	3.39E-06	14.9639	9.29548	0.987752	9.41074
CEU;Xun	0.00005066	3.48E-06	14.5556	9.29548	0.987752	9.41074
GBR;GuiGhanaKgal	0.00005041	3.38E-06	14.9277	9.29548	0.987752	9.41074
IBS;Juhoansi	0.00005039	3.16E-06	15.9483	9.29548	0.987752	9.41074
CEU;GuiGhanaKgal	0.00005036	3.47E-06	14.5179	9.29548	0.987752	9.41074
Juhoansi;TSI	0.00005016	3.15E-06	15.9389	9.29548	0.987752	9.41074
FIN;Xun	0.00004969	3.23E-06	15.3798	9.29548	0.987752	9.41074
FIN;GuiGhanaKgal	0.00004941	3.18E-06	15.5391	9.29548	0.987752	9.41074
IBS;Xun	0.00004789	3.10E-06	15.4348	9.29548	0.987752	9.41074
TSI;Xun	0.00004769	3.09E-06	15.4221	9.29548	0.987752	9.41074
GuiGhanaKgal;IBS	0.00004766	3.01E-06	15.859	9.29548	0.987752	9.41074
GuiGhanaKgal;TSI	0.00004746	2.99E-06	15.857	9.29548	0.987752	9.41074
GBR;SEBantu	0.00004626	3.12E-06	14.8489	9.29548	0.987752	9.41074
CEU;SEBantu	0.00004622	3.20E-06	14.4466	9.29548	0.987752	9.41074
GBR;YRI	0.00004565	3.08E-06	14.8185	9.29548	0.987752	9.41074
CEU;YRI	0.00004557	3.16E-06	14.4325	9.29548	0.987752	9.41074
FIN;SEBantu	0.00004533	2.94E-06	15.4212	9.29548	0.987752	9.41074
GBR;Khwe	0.00004517	3.03E-06	14.8971	9.29548	0.987752	9.41074
CEU;Khwe	0.00004511	3.11E-06	14.494	9.29548	0.987752	9.41074
FIN;YRI	0.00004475	2.91E-06	15.3868	9.29548	0.987752	9.41074
FIN;Khwe	0.00004434	2.87E-06	15.4744	9.29548	0.987752	9.41074
IBS;SEBantu	0.00004366	2.77E-06	15.7707	9.29548	0.987752	9.41074
SEBantu;TSI	0.00004350	2.76E-06	15.743	9.29548	0.987752	9.41074

Note: certain populations had to be excluded from the analyses (e.g. Khomani and Karretjie people) because of long-range LD blocks in these populations.

Table S5: Top 5 peaks detected with Locus Specific Branch Length scans (indicating allele frequency differences of AFR compared to CEU and GBR)

**Peak**	**Position information (chr:hg19 chr position)**	**Standard Deviations from the mean**	**Underlying or nearby genes***	**Potential function of underlying/nearby genes***
LSBL-1	17:19990968	19.1	*SPECC1*	The protein encoded by this gene belongs to the cytospin-A family. It is localized in the nucleus, and highly expressed in testis and some cancer cell lines. A chromosomal translocation involving this gene and platelet-derived growth factor receptor, beta gene (*PDGFRB*) may be a cause of juvenile myelomonocytic leukemia. Alternatively spliced transcript variants encoding different isoforms have been described for this gene. [provided by RefSeq, Aug 2011]
LSBL-2	2:169122966	16.3	Closest gene is *STK39* peak SNP is 18 kb upstream from the gene	This gene encodes a serine/threonine kinase that is thought to function in the cellular stress response pathway. The gene has ubiquitous expression, with most abundant expression in brain and pancreas. The kinase is activated in response to hypotonic stress, leading to phosphorylation of several cation-chloride-coupled cotransporters. The catalytically active kinase specifically activates the p38 MAP kinase pathway, and its interaction with p38 decreases upon cellular stress, suggesting that this kinase may serve as an intermediate in the response to cellular stress. [provided by RefSeq, Jul 2008]
LSBL-3	21:40033056	14.4	*ERG*	This gene encodes a member of the erythroblast transformation-specific (ETS) family of transcriptions factors. All members of this family are key regulators of embryonic development, cell proliferation, differentiation, angiogenesis, inflammation, and apoptosis. The protein encoded by this gene is mainly expressed in the nucleus. It contains an ETS DNA-binding domain and a PNT (pointed) domain which is implicated in the self-association of chimeric oncoproteins. This protein is required for platelet adhesion to the subendothelium, inducing vascular cell remodeling. It also regulates hematopoesis, and the differentiation and maturation of megakaryocytic cells. This gene is involved in chromosomal translocations, resulting in different fusion gene products, such as TMPSSR2-ERG and NDRG1-ERG in prostate cancer, EWS-ERG in Ewing's sarcoma and FUS-ERG in acute myeloid leukemia. More than two dozens of transcript variants generated from combinatorial usage of three alternative promoters and multiple alternative splicing events have been reported, but the full-length nature of many of these variants has not been determined. [provided by RefSeq, Apr 2014].
LSBL-4	9:127224330	13.6	*GPR144*	Homo sapiens G protein-coupled receptor 144 (*GPR144*), mRNA
LSBL-5	5:125538397	13.5	*RP11-114J13.1*	lincRNA, no known function.

*Underlying/nearby gene information and gene functions were obtained from UCSC genome browser (https://genome.ucsc.edu/)

Table S6: Top 5 selection scan peaks detected with iHS and XP-EHH scans

**Peak**	**Position information** (chr:hg19 chr position)	**Standard Deviations from the mean**	**Underlying or nearby genes***	**Potential function of underlying/nearby genes***
iHS-1	18:7486106	17.9	No underlying genes, Closest gene: *PTPRM* (top SNP in peak is 82 kb upstream from gene)	*PTPRM*: member of the protein tyrosine phosphatase (PTP) family. PTPs are known to be signaling molecules that regulate a variety of cellular processes including cell growth, differentiation, mitotic cycle, and oncogenic transformation
iHS-2	9:90898783	17.2	No underlying genes, Closest gene: *SPIN1* (top SNP in peak is 143 kb upstream from gene)	Homo sapiens spindlin 1 (*SPIN1*), mRNA. No known function
iHS-3	13:45630008	14.3	No underlying genes, Closest gene: *KIAA1704* (top SNP in peak is 41 kb downstream from gene)	Homo sapiens *KIAA1704* (KIAA1704), mRNA. No known function
iHS-4	4:123774065	14.1	Underlying gene: *FGF2*	Homo sapiens fibroblast growth factor 2 (*FGF2*). The protein encoded by this gene is a member of the fibroblast growth factor (FGF) family. FGF family members bind heparin and possess broad mitogenic and angiogenic activities. This protein has been implicated in diverse biological processes, such as limb and nervous system development, wound healing, and tumor growth. Positive Disease Associations: Cholesterol, LDL [PubMed 17903299]
iHS-5	17:36185998	13.1	No underlying genes, Closest genes are:<br><br>*LOC284100* (top SNP in peak is 17 kb downstream from gene)<br><br>*HNF1B*: (top SNP in peak is 139 kb upstream from gene)	*LOC284100*: Homo sapiens tyrosine 3-monooxygenase/tryptophan 5-monooxygenase activation protein, epsilon polypeptide pseudogene (LOC284100), non-coding RNA. No known function<br><br>*HNF1B*: Homo sapiens HNF1 homeobox B. This gene encodes a member of the homeodomain-containing superfamily of transcription factors. The protein binds to DNA as either a homodimer, or a heterodimer with the related protein hepatocyte nuclear factor 1-alpha. The gene has been shown to function in nephron development, and regulates development of the embryonic pancreas. Mutations in this gene result in renal cysts and diabetes syndrome and noninsulin-dependent diabetes mellitus, and expression of this gene is altered in some types of cancer.

XP-EHH-1	18:57128753	8.8	Underlying gene: *CCBE1*	Homo sapiens collagen and calcium binding EGF domains 1 (*CCBE1*). This gene is thought to function in extracellular matrix remodeling and migration. It is predominantly expressed in the ovary, but down regulated in ovarian cancer cell lines and primary carcinomas, suggesting its role as a tumour suppressor. Mutations in this gene have been associated with Hennekam lymphangiectasia-lymphedema syndrome, a generalized lymphatic dysplasia in humans.<br>Positive Disease Associations: Alcoholism, Apolipoproteins B, Arteries, Blood Pressure Determination, Body Mass Index, Cell Adhesion Molecules, Cholesterol, Insulin, Rheumatoid Arthritis
XP-EHH-2	3:18609478	6.6	No underlying genes, Closest gene:<br><br>*SATB1* (top SNP in peak is 220 kb upstream from gene)	Homo sapiens SATB homeobox 1 (*SATB1*). This gene encodes a matrix protein which binds nuclear matrix and scaffold-associating DNAs through a unique nuclear architecture. The protein recruits chromatin-remodeling factors in order to regulate chromatin structure and gene expression. Crucial silencing factor contributing to the initiation of X inactivation mediated by Xist RNA that occurs during embryogenesis and in lymphoma. Positive Disease Associations: Alcoholism, Arteries, Cholesterol, HDL, Crohn Disease, Eosinophils, Inflammatory Bowel Diseases, Insulin, Insulin Resistance, Psoriasis
XP-EHH-3	2:74122223	6.3	Underlying gene: *ACTG2*	Homo sapiens actin, gamma 2, smooth muscle, enteric (*ACTG2*). Actins are highly conserved proteins that are involved in various types of cell motility and in the maintenance of the cytoskeleton. This gene encodes actin gamma 2; a smooth muscle actin found in enteric tissues. Diseases sorted by gene-association score: visceral myopathy* (1599), chronic intestinal pseudoobstruction* (400), myopathic intestinal pseudoobstruction* (350), actg2 (20), intestinal pseudo-obstruction (13), peyronie's disease (8), myopathy (3)
XP-EHH-4	3:67456973	6.0	Underlying gene: *SUCLG2*	Homo sapiens succinate-CoA ligase, GDP-forming, beta subunit (*SUCLG2*), nuclear gene encoding mitochondrial protein. This gene encodes a GTP-specific beta subunit of succinyl-CoA synthetase. Succinyl-CoA synthetase catalyzes the reversible reaction involving the formation of succinyl-CoA and succinate. Positive Disease Associations: Astigmatism , Glucose , Lipoproteins, VLDL
XP-EHH-5	6:128989302	5.7	No underlying genes, Closest gene:	Homo sapiens laminin, alpha 2 (*LAMA2*). Laminin, an extracellular protein, is a major component of the basement membrane. It is thought to mediate the attachment, migration, and organization of cells into tissues during embryonic development by

			LAMA2 (top SNP in peak is 215 kb upstream from gene)	interacting with other extracellular matrix components. Mutations in this gene have been identified as the cause of congenital merosin-deficient muscular dystrophy. Positive Disease Associations: Amyotrophic Lateral Sclerosis, Body Mass Index, Body Weight, Cholesterol, HDL, Echocardiography, Exercise Test, leprosy, tuberculoid type of leprosy, Uric Acid

*Underlying/nearby gene information and gene functions were obtained from UCSC genome browser (https://genome.ucsc.edu/)

Supplementary Text

This supplementary note discusses the populations from which the Afrikaner population arose, it summarizes genealogical information of admixture and presents genetic information of admixture.

Population descriptions

1. Khoekhoe and San

The population of Khoekhoe and San, hereafter referred to as Khoe-San is estimated to have numbered 20,000 overall and 5,000 people in the western Cape in 1652 [3]. Annexation of Khoe-San grazing lands, direct conflicts and raids of their life stock by Afrikaner frontier farmers gradually forced them out of the Western Cape [3]. The small pox epidemic of 1713 was particularly harsh on the Khoe-San population with only 1 in 10 surviving. After the epidemic there were very few Khoe-San individuals left in the Western Cape [3]. Continual wars against the Khoe-San, systematic killing of the Khoe-San, and capturing of Khoe-San women and children as so-called "inboekelinge" (to force them into a serfdom/indentured labour system) continued to reduce their numbers in areas occupied by Afrikaner frontier farmers [3-5].

2. European colonists

Europeans started independent settlement at the Cape from 1658. Since they were to some extent independent of the Dutch East Indian Company (DEIC) they were initially called "free citizens" and as the populations' identity evolved they were referred to as Afrikaners (although "Africander" was initially used to indicate locally born slaves). The population had a high growth rate of about 2.6% per annum [6]. Although a small part of this increase was the result of a continuous arrival of immigrants, it is mostly due to the population's high fecundity (Figure S12).

The immigrants from Europe originated from many different countries and the DIC encouraged immigration by offering free passage to the Cape and granting land for farming [7]. The Netherlands, Germany and France were the most important sources of immigrants with them respectively donating 34%-37%, 27%-34% and 13%-26% of the immigrants.

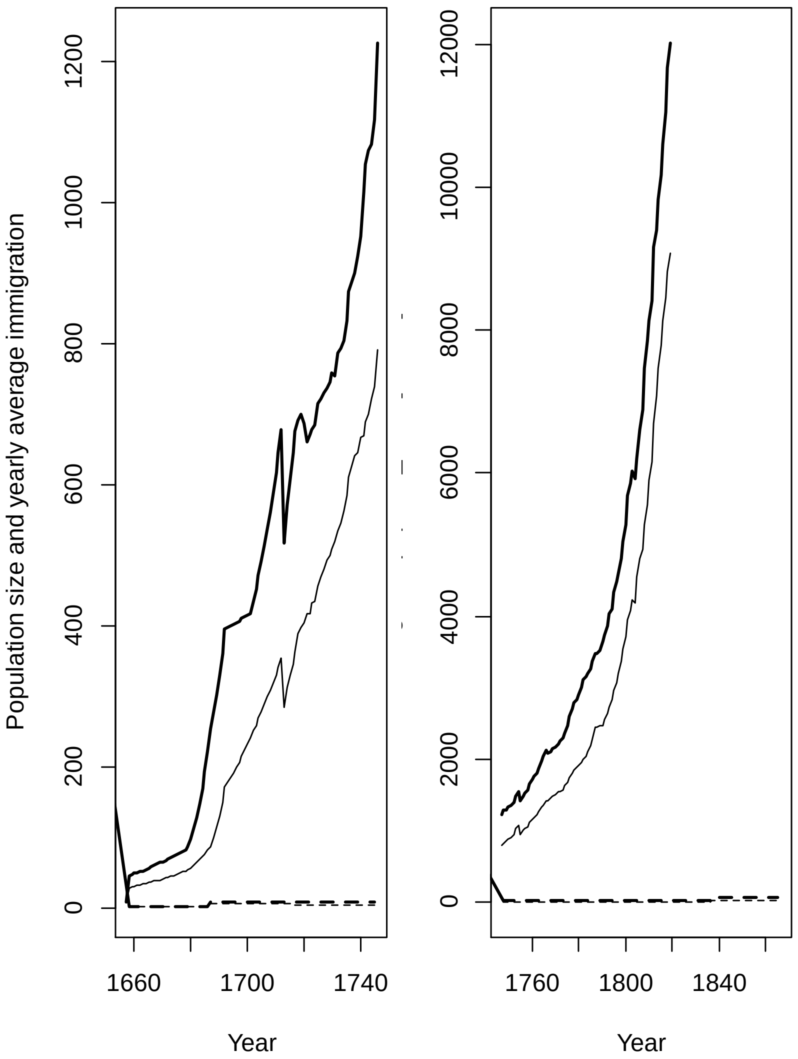

Figure S12. The population size of adult Afrikaner men (solid heavy line) and women (solid lighter line) as a function of time. Data was obtained from Gouws [8]. The yearly average number of immigrants that married into Afrikaner families is given in dashed lines with men in heavy and women in lighter lines. Data was counted from Heese [9]. These values are averages for 30 year periods, so for instance, from 1808 to 1837, 970 men who were not born at the Cape, got married in the colony, giving a yearly rate of 32⅓. Note the different scales on the y-axes. The Y-axis in the second panel starts where the axis ends in the first panel.

3. Slaves

Starting with two boats carrying about 400 slaves from West Africa in 1658 the slave population grew to 36,169 in 1834 when slavery was abolished at the Cape [10]. The total number of slaves and their origin has to be inferred indirectly, because records are missing and many arrivals were not recorded (Worden pers comm). Even so, Shell [4] argued that about 63,000 slaves arrived in the colony between 1658-1807. He calculated that roughly a quarter came from each of; Africa

(26.4%, east coast, excepting first two shiploads), Madagascar and Mascarenes (25.1%), South Asia (25.9%) and Southeast Asia (22.7%) [4]. Based on household inventories, Worden [11, 12] estimated that more slaves came from Asia, especially South Asia, and that substantially fewer came from Madagascar and Africa. Both sources suggest marked temporal variation in the origin of slaves, with Asian slaves predominating in the 17th and earlier 18th centuries and African slaves dominating towards 1807 (Figure S13). Initially the DEIC owned most slaves but after 1692 colonists owned the majority [10]. Shortly after 1710 slaves outnumbered the colonists and this situation remained until the abolition of slave trade in 1807 [10]. Although the slave population creolized (>50% locally born) in 1770 [4], the local growth was lower than that of the colonists [10]. After emancipation in 1834 slaves were freed after a four-year apprenticeship and formed the backbone of the Coloured community.

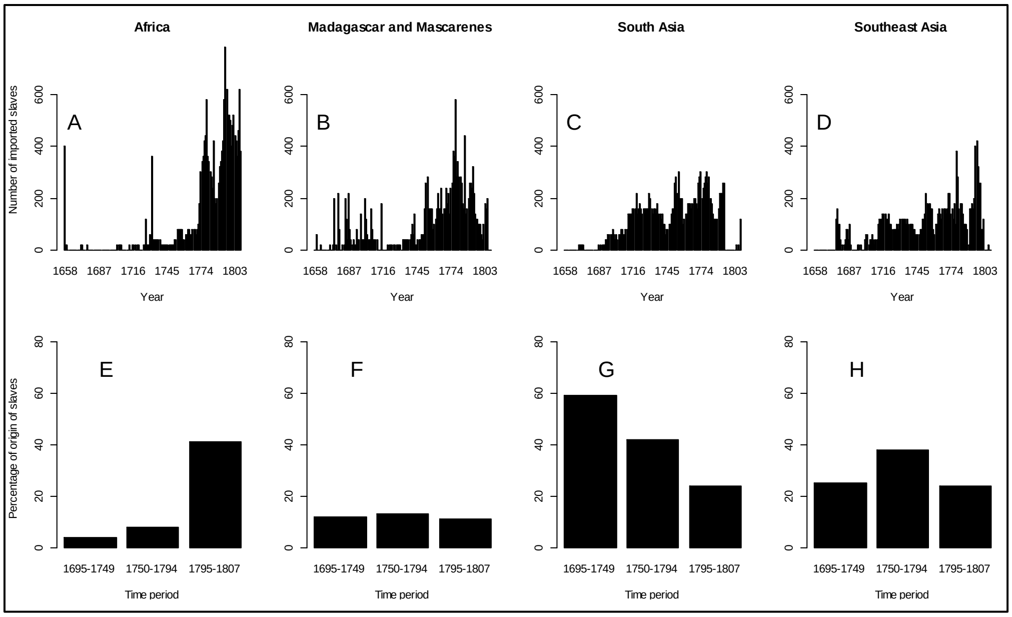

Figure S13. The origins of slaves arriving in the Cape. Estimates based on Shell [4] (A-D) and Worden [11, 12] (E-H). Shell's [4] data were read off his figure 2-1, rounded to the nearest 20, with slight adjustments to 1) fit the time line of arrivals from 1658 – 1807, and to 2) correct for the first two vessels that arrived from Western Africa in 1658. Worden's [11, 12] estimates come from his Appendix. Slaves were from Africa (A & E), Madagascar and Mascarenes (B & F), South Asia (C and G) and Southeast Asia (D & H).

4. *Absence of South African Bantu-speakers*

Bantu-speakers originated from West Africa and started to expand to the rest of sub-Saharan Africa around 4,000 years ago, reaching the northern parts of current-day South Africa around 1,800 years ago. During the 1600s the edge of the Bantu-expansion (specifically Xhosa speakers) reached as far south as the Fish River in the Eastern Cape Province of current day South Africa (approximately 1000 km from Cape Town). Therefore, Bantu-speakers were only encountered at a much later stage during the frontier wars in 1779-1851 by expanding Afrikaner frontier farmers [13].

Although initial interactions between the frontier farmers and Bantu-speaking Xhosa populations were peaceful with trade between groups, relations quickly turned aggressive and conflicts erupted. It is unlikely that much gene flow occurred between South African Bantu-speakers and Afrikaners, since during this relatively late time period, the Afrikaner population identity had already culminated, which resulted in social hinders to relationships between groups.

Genealogical evidence of admixture

Historians in South Africa compiled genealogical registries for the Afrikaner population based on church records (summarized in Greeff & Erasmus [14]). From these records it is clear that the Afrikaner population is admixed (Table S7). The estimates vary, but the non-European contribution is between 5.4 and 7.2 percent.

While many unions between slaves were recorded in church records, only one instance of a union between a European and Khoe-San was solemnized at the Cape [3]. People that are given the toponym "van die Kaap" (meaning from the Cape) rather than a surname are of uncertain heritage. While these individuals were born at the Cape their parent(s) may have been a slave, Khoe-San or even European [15]. For this reason and for other reasons given in the main text, a genetic analysis is required to make an accurate calculation of the population's constitution.

Genetic structure of the Afrikaner population

Surprisingly little is known about the genetic structure of the Afrikaner population. The few genetic studies on Afrikaners suggested admixture between European and non-European populations. 1) For some loci Afrikaners have more alleles than Europeans [16] and 2) Afrikaners have non-European alleles at some loci [17]. Botha & Pritchard [18] estimated that slaves from Africa and Asia, and/or Khoe-San, contributed between 6 – 7% of genes to the Afrikaner population. They based their estimate on blood group allele frequencies.

A number of familial diseases are more common in Afrikaners than in European populations [19-27]. These data supports the notion of a significant founder effect during the formation of the population [17, 28, 29]. While a deep routing pedigree of one Afrikaner clearly illustrated the existence of many common ancestors, these were so distant that his pedigree-inbreeding coefficient was not higher than European averages [30].

Table S7. Four estimates from three studies of the percentage composition of Afrikaners.

Origin of contributions	All immigrants up to 1867 weighted by entry time[a]	All immigrants up to 1807 weighted by entry time[a]	1063 baptisms in 1807 in Cape[b]	One living individual[c]
**European**	**89.6[d]**	**89.3**	**90.7**	**93.34**
Netherlands	34.8	36.8	34.1	37.22
German	33.7	35.0	29.2	27.21
French	13.2	14.6	24.7	26.26
Other European	7.9	2.9	2.7	2.65
**Non-Euroean**	**6.9**	**7.2**	**5.4**	**6.04**
East-Asian				1.89
South-East Asian				0
South Asian				1.66
India				1.66
Africa				2.48
Madagaskar				0.06
Guinea				0.27
Van die Kaap[e]				2.15
**Unknown**	**3.5**	**3.5**	**3.9**	**0.62**

[a] Heese [9] divided 1657-1867 into seven 30-year slots (and for the period 1657-1807 in 5 30-year slots), for each slot new immigrants are listed and their contribution was weighted by their number of children and each earlier time-slot was weighed twice as heavily as the subsequent time-slot. Hence, an immigrant in 1658 contributed 64 “blood units” per child whereas an individual in 1840 contributed 1 per child.
[b] de Bruyn [31] quantified the average composition of 1063 children baptized in the Cape in 1807. This sample represents about 15% of the entire Afrikaner population at the time.
[c] Percentages divided by 82.81 from Greeff [30]. It corrects for incomplete lineages.
[d] numbers in bold are totals of normal type indented from each area listed underneath.
[e] The expression “van die Kaap” was given as a toponymical surname and means “from the Cape”. It was given to children born at the Cape and was presumably used to hide the fact that their parents were slaves or manumitted slaves. However, their parents may have been Khoe-San or even European [15].

Supplementary References

3. Elphick R, Malherbe VC: **The Khoisan to 1828**. In: *The Shaping of South African Society, 1652-1840.* Edited by Elphick R, Giliomee H, 2nd edn. Cape Town: Maskew Miller Longman (Pty) Ltd; 1989: 3-65.
4. Shell RCH: **Children of bondage: A social history of the slave society at the Cape of Good Hope, 1652-1838**: Wesleyan University Press published by University Press of New England; 1994.

5. Terreblanche S: **A history of inequality in South Africa, 1652-2002**. Pietermaritzburg: University of Natal Press; 2002.
6. Ross R: **The “white” population of the Cape colony in the eighteenth century**. In: *Beyond the Pale*. Edited by Ross R: Wesleyan University Press; 1993: 125-137.
7. Guelke L: **Freehold farmers and frontier settlers, 1657-1780**. In: *The Shaping of South African Society, 1652-1840*. Edited by Elphick R, Giliomee H, 2nd edn. Cape Town: Maskew Miller Longman (Pty) Ltd; 1989: 66-108.
8. Gouws NB: **Bevolkingstendense van Blankes in Suid-Afrika voor 1820**. Johannesburg: Randse Afrikaanse Universiteit; 1981.
9. Heese JA: **Die Herkoms van die Afrikaner, 1657–1867**. Kaapstad: A.A. Balkema; 1971.
10. Armstrong JC, Worden NA: **The slaves, 1657-1834**. In: *The Shaping of South African Society, 1652-1840*. Edited by Elphick R, Giliomee H, 2nd edn. Cape Town: Maskew Miller Longman (Pty) Ltd; 1989: 109-183.
11. Worden N: **Indian Ocean slaves in Cape Town, 1695 – 1807**. *Journal of South African Studies* 2016, **42**(3):389-408.
12. Worden N: **The changing nature of Indian Ocean slave trading to the Cape colony, 1652 – 1807. Slave Trade in the Indian Ocean and Indonesian Archipelago Worlds (16th to 19th Century)**. In: *New Research, Results and Comparisons International Workshop (10-11 Nov 2016): 2016; IISH, Amsterdam*; 2016.
13. Giliomee H, Mbenga B: **New History of South Africa**. Cape Town: Tafelberg; 2007.
14. Greeff JM, Erasmus JC: **Three hundred years of low non-paternity in a human population**. *Heredity (Edinb)* 2015, **115**(5):396-404.
15. **Who was Susanna Claassen? Accessed 2018.0508** [http://www.eggsa.org/articles/SusannaClaasz/SusannaClaasz.htm]
16. Erasmus JC, Klingenberg A, Greeff JM: **Allele frequencies of AVPR1A and MAOA in the Afrikaner population**. *S Afr J Sci* 2015, **111**:114-119.
17. Nurse GT, Weiner JS, Jenkins T: **The Peoples of Southern Africa and their Affinities**. New York: Oxford University Press; 1985.
18. Botha MC, Pritchard J: **Blood group gene frequencies: an indication of the genetic constitution of population samples in Cape Town**. *S Afr Med J* 1972, **46**:S1–27.
19. Dean G: **The Porphyrias: a story of inheritance and environment**. Bath: The Pitman Press; 1963.
20. Cilliers HJ, Beighton P: **Beukes familial hip dysplasia: an autosomal dominant entity**. *Am J Med Genet* 1990, **36**(4):386-390.
21. Jenkins T, Nicholls E, Gordon E, Mendelsohn D, Seftel HC, Andrew MJ: **Familial hypercholesterolaemia--a common genetic disorder in the Afrikaans population**. *S Afr Med J* 1980, **57**(23):943-947.
22. Hayden MR, Hopkins HC, Macrea M, Beighton PH: **The origin of Huntington's chorea in the Afrikaner population of South Africa**. *S Afr Med J* 1980, **58**(5):197-200.
23. Rosendorff J, Bernstein R, Macdougall L, Jenkins T: **Fanconi anemia: another disease of unusually high prevalence in the Afrikaans population of South Africa**. *Am J Med Genet* 1987, **27**(4):793-797.
24. Torrington M, Viljoen DL: **Founder effect in 20 Afrikaner kindreds with pseudoxanthoma elasticum**. *S Afr Med J* 1991, **79**(1):7-11.

25. Torrington M, Weymar HW, van der Merwe PL, Brink AJ: **Progressive familial heart block. Part I. Extent of the disease**. *S Afr Med J* 1986, **70**(6):353-355.
26. Heyl T: **Genealogical study of lipoid proteinosis in South Africa**. *Br J Dermatol* 1970, **83**(3):338-340.
27. Beighton P, Davidson J, Durr L, Hamersma H: **Sclerosteosis - an autosomal recessive disorder**. *Clin Genet* 1977, **11**(1):1-7.
28. Botha MC, Beighton P: **Inherited disorders in the Afrikaner population of southern Africa. Part I. Historical and demographic background, cardiovascular, neurological, metabolic and intestinal conditions**. *S Afr Med J* 1983, **64**(16):609-612.
29. Botha MC, Beighton P: **Inherited disorders in the Afrikaner population of southern Africa. Part II. Skeletal, dermal and haematological conditions; the Afrikaners of Gamkaskloof; demographic considerations**. *S Afr Med J* 1983, **64**(17):664-667.
30. Greeff JM: **Deconstructing Jaco: genetic heritage of an Afrikaner**. *Ann Hum Genet* 2007, **71**(Pt 5):674-688.
31. de Bruyn GFC: **Die samestelling van die Afrikanervolk. [The composition of the Afrikaner population]**. *Tydskrif vir Geesteswetenskap* 1976, **15**:39–42.
